# Conserved role of FOXC1 in TNBC is parallel to FOXA1 in ER+ breast cancer

**DOI:** 10.1016/j.isci.2024.110500

**Published:** 2024-07-14

**Authors:** Revathy Ramachandran, Shakhzada Ibragimova, Laura M. Woods, Tamader AlHouqani, Roshna Lawrence Gomez, Fabrizio Simeoni, Mahmood Y. Hachim, Tim C.P. Somervaille, Anna Philpott, Jason S. Carroll, Fahad R. Ali

**Affiliations:** 1 College of Medicine, Mohammed Bin Rashid University of Medicine and Health Sciences, Dubai, United Arab Emirates; 2 Wellcome-MRC Cambridge Stem Cell Institute, Jeffrey Cheah Biomedical Centre, Cambridge Biomedical Campus, Cambridge, UK; 3 Department of Oncology, University of Cambridge, Cambridge, UK; 4 Cancer Research UK Cambridge Institute, University of Cambridge, Cambridge, UK; 5 Cancer Research UK Manchester Institute, University of Manchester, Manchester, UK

**Keywords:** Molecular biology, Cancer, Omics, Proteomics, Transcriptomics

## Abstract

Triple-negative breast cancer (TNBC) is characterized by lack of the estrogen (ER) receptor, progesterone receptor (PR), and human epidermal growth factor receptor-2 (HER2), and standard receptor-targeted therapies are ineffective. FOXC1, a transcription factor aberrantly overexpressed in many cancers, drives growth, metastasis, and stem-cell-like properties in TNBC. However, the molecular function of FOXC1 is unknown, partly due to heterogeneity of TNBC. Here, we show that although FOXC1 regulates many cancer hallmarks in TNBC, its function is varied in different cell lines, highlighted by the differential response to CDK4/6 inhibitors upon FOXC1 loss. Despite this functional heterogeneity, we show that FOXC1 regulates key oncogenes and tumor suppressors and identify a set of core FOXC1 peaks conserved across TNBC cell lines. We identify the ER-associated and drug-targetable nuclear receptor NR2F2 as a cofactor of FOXC1. Finally, we show that core FOXC1 targets in TNBC are regulated in parallel by the pioneer factor FOXA1 and the nuclear receptor NR2F2 in ER + breast cancer.

## Highlights

• FOXC1 regulates oncogenic phenotypes in TNBC via varied gene targets
• We have identified core FOXC1 gene targets conserved across TNBC cell lines
• FOXC1 interacts with nuclear receptor NR2F2
• Core FOXC1 targets in TNBC are regulated by FOXA1 in luminal breast cancer

## Graphical abstract

**Figure:**
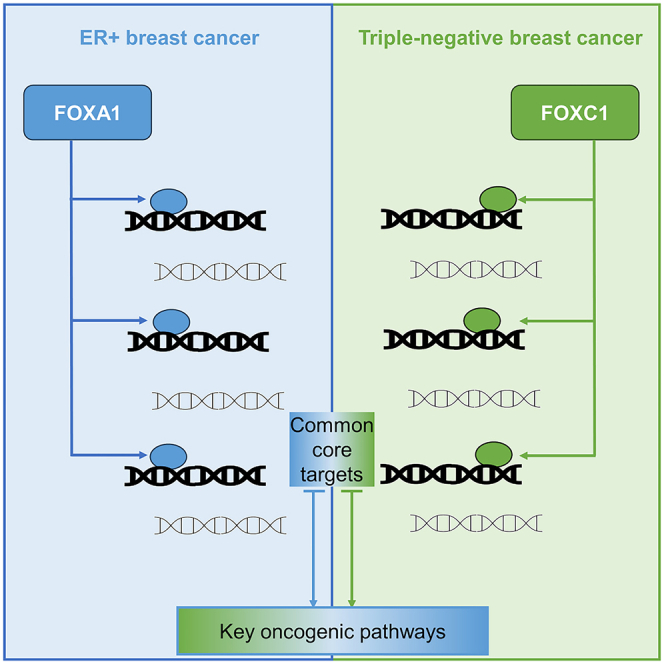

## Introduction

Triple-negative breast cancer (TNBC) is defined by the absence of the estrogen receptor (ERα), progesterone receptor (PR), and the human epidermal growth factor receptor-2 (ERBB2/HER2) in tumor cells. TNBC accounts for around ∼15% of all diagnosed breast cancer cases and is aggressive, prone to metastasis, and has few targeted therapies available.^1^^,^^2^ A compounding issue in TNBC treatment is its heterogeneous nature, reflected in the high variability of TNBC cell line models.^3^ Sub-classification of TNBC based on transcriptional or molecular properties has been attempted, mainly to identify responsiveness to different therapies and afford patients the best available treatment options.^4^ In the absence of hormone receptor-driven growth signaling, it is unclear what drives the proliferation of tumor cells in TNBC.^5^ This is in contrast to the luminal breast cancer subtype, where the transcriptional cascade spearheaded by ERα and its co-factors stimulates proliferative pathways and are thus targeted by therapies.^6^

Crucial to the development of targeted therapies in TNBC is the identification of key drivers of proliferation and their detailed molecular function. Transcription factor (TF) FOXC1 is over-expressed in TNBC and has diagnostic and prognostic value, with its expression correlating with chemoresistance and metastasis in basal-like breast cancer.^7^^,^^8^^,^^9^^,^^10^ Over-expression of FOXC1 in TNBC cell lines has generally been shown to increase tumorigenic properties such as proliferation, invasion, and migration,^9^^,^^11^^,^^12^ although one study reported inhibition of metastasis upon over-expression of FOXC1.^13^ FOXC1 belongs to the human Forkhead family of around 50 TFs that contain the highly conserved Forkhead DNA binding domain. It is expressed during embryogenesis and is crucial for mesenchymal lineage specification and organ development, including bone, cartilage, and eye.^14^ FOXC1 is overexpressed in many cancers such as breast, bladder, colorectal, acute myeloid leukemia (AML), hepatocellular carcinoma, and non-small cell lung cancer^10^ and is a patented clinical marker for TNBC, particularly for the basal-like phenotype.^7^^,^^15^ FOXC1 has been shown to be involved with epidermal growth factor receptor (EGFR) function, NF-κB, canonical and non-canonical Wnt, PI3K/Akt/mTOR, and non-canonical Hedgehog signaling (reviewed in a study by Ray et al.^10^).

Nonetheless, genome-wide analysis of FOXC1 binding sites and identification of FOXC1-regulated genes in TNBC has not been reported. Furthermore, there is variation in expression of the FOXC1 protein among TNBC cell lines, and it remains unknown whether the level of FOXC1 protein expression correlates with its function. Since TNBC is a heterogeneously classified subgroup of breast cancer, the identification of core conserved FOXC1-regulated gene networks will elucidate the functional role of FOXC1 in TNBC and could improve our understanding of TNBC pathogenesis. In this study, using transcriptomic, epigenetic, proteomic, and bioinformatic analysis, we aim to identify an exhaustive list of FOXC1 targets and cofactors and define, in greater detail, its conserved molecular function across breast cancer.

## Results

### FOXC1 regulates oncogenic phenotypes via varied gene targets in TNBC

**Figure 1:**
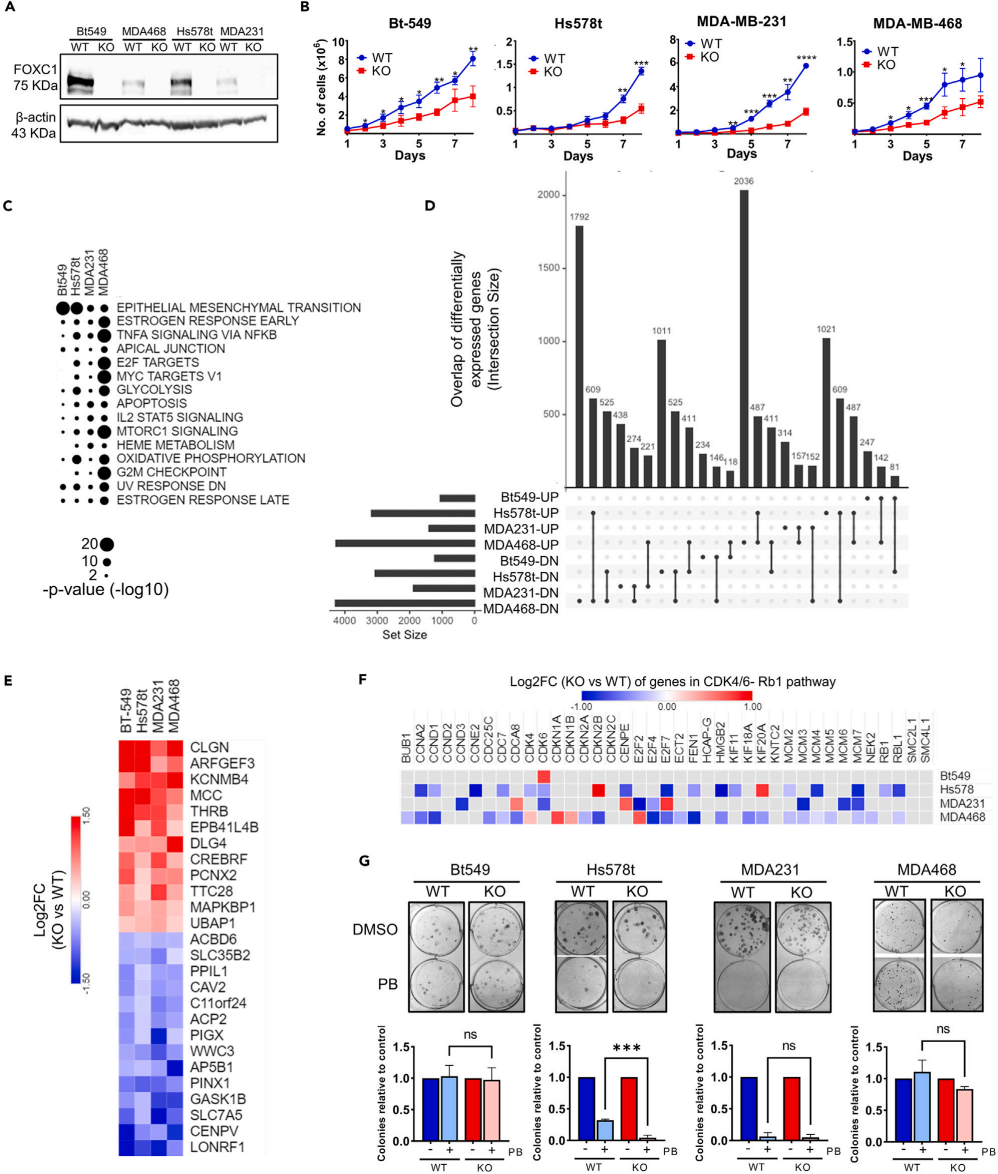
Effect of FOXC1 knockout on phenotype and gene expression (A) Western blot of cell lysate (75 μg) from four parental TNBC cell lines (“WT”) and the corresponding FOXC1 knockout CRISPR clones (“KO”), probed using anti-FOXC1 and anti-β-actin antibodies. (B) Proliferation assays showing growth of FOXC1_KO clones (red) when compared to the parental cell lines (blue). Viable cells were counted every 24 h. Data represent mean and standard deviation (SD) of three biological replicates. Asterisk denotes significant digits in *p* value derived from unpaired t tests with ∗*p* < 0.05, ∗∗*p* < 0.005, and ∗∗∗*p* < 0.0005. (C) Enrichment using Hallmark gene sets (MSigDB^16^) showing significantly enriched (padj < 0.01) cancer hallmarks among the DEGs in each of the four TNBC cell lines. (D) Upset plot depicting intersection between up- or downregulated genes among the four cell lines, showing a limited set of DEGs that overlap and are similarly regulated (up- or down-) in all four cell lines. (E) Heatmap depicting log2 (fold change) of the 26 genes significantly (padj < 0.05) regulated upon the loss of FOXC1 in all four cell lines, representing genes repressed by FOXC1 (red) and genes activated by FOXC1 (blue). (F) Log2 (fold change) of selected genes in the RB1/CDK4/6 pathway (FOXC1_KO vs. WT). (G) Colony-forming assays performed in 6-wells in the presence of either DMSO (top row) or 0.5 μM palbociclib (bottom row) for 14 days prior to crystal violet staining. Bar graphs (bottom panel) display relative reduction in the number of colonies compared to the DMSO control in the parental (WT, blue) or the FOXC1_KO (KO, red) groups. Data represents mean ± SD from three biological replicates each. Asterisk denotes significant digits in *p* value derived from unpaired t tests. ∗∗∗*p* < 0.005.

To study the function of FOXC1 in TNBC, we generated CRISPR-edited FOXC1 gene knockouts (FOXC1_KO) in four TNBC cell lines: BT-549, Hs578t, MDA-MB-231, and MDA-MB-468 (Table S1). The cell lines were selected to represent variation in FOXC1 protein expression, with BT-549 and Hs578t expressing higher amounts (Figure 1A). Homozygous clones successfully edited for FOXC1_KO were verified using Sanger sequencing, and absolute loss of protein expression was confirmed using western blot (Figure 1A).

Upon loss of FOXC1, growth of the FOXC1_KO clones was slower than their respective parental cells (Figure 1B). Morphological changes were observed in all cell lines, with the FOXC1_KO clones adopting a flatter, cuboid appearance with decreased cell-cell adhesion and in the case of Hs578t, losing the mesenchymal morphology altogether (Figure S1A). Invasion, migration, and colony forming assays corroborate the role of FOXC1 as an oncogene that regulates proliferative, migratory, invasive, and clonogenic properties in TNBC, although the percent reduction in oncogenic phenotypes varied among cell lines and did not correlate with the FOXC1 protein expression levels (Figures S1B and S1C). The cell cycle profile between the parental and FOXC1_KO clones was not significantly altered, except in the case of Hs578t, where a higher proportion of cells were present in the S-phase in FOXC1_KO (Figure S1D), suggesting a cell line specific function of FOXC1.

In order to identify common genes and pathways regulated by FOXC1 across TNBC, RNA sequencing (RNA-seq) analysis was performed on the four parental TNBC cell lines (“WT”) and matched FOXC1-deleted CRISPR clones (“KO”). Principal component analysis (PCA) and correlation of gene expression profiles show that the cell line accounts for the greatest source of the variation in the dataset, with a smaller effect of FOXC1 status (Figures S2A and S2B, respectively). Differentially expressed genes (DEGs) were identified using filtering criteria (false discovery rate [FDR] < 0.05) and no correlation was observed between the number of DEGs and FOXC1 protein expression, as both BT-549 and MDA-MB-231 had a similar number of DEGs even though BT-549 has higher FOXC1 protein expression (Figure S2C). Enrichment of pathways in each individual cell line revealed regulation of major cancer hallmarks such as epithelial to mesenchymal transition, apoptosis, hypoxia, and hormone response (Figure 1C). However, upset plots show a limited set of DEGs that overlap and are similarly regulated (up- or down-) in all four cell lines (Figure 1D). Although 172 genes were significantly (*p*adj value < 0.05) differentially expressed between WT and FOXC1_KO, of the four cell lines (Figure S2D), only 26 genes were similarly regulated (either up- or downregulated across all four cell lines); these 26 genes represent a high stringency list of the FOXC1 regulon in TNBC (Figure 1E). Unsupervised k-means clustering employed to the 4 WT vs. KO pairs generated 13 clusters based on the pattern of gene expression (gene expression patterns, Figure S2E; heatmap, Figure S2F). Enrichment of Gene Ontology (GO) terms biological processes reveal downregulation of DNA replication upon loss of FOXC1 in cluster 8 (similarly downregulated genes) and no significantly enriched terms in the cluster 13 (upregulated genes) (Figure S2G). The lack of majority in either cluster suggests that the functional FOXC1 regulon in TNBC is largely cell-line dependent. Although FOXC1 has been implicated in the silencing of ERα,^17^ no significant change in *ESR1* gene expression was observed (Figure S2H).

In Hs578t, MDA-MB-231, and MDA-MB-468, targets of E2F were significantly enriched (Figure 1C), and many genes involved in the RB1 pathway were differentially expressed upon loss of FOXC1, except in BT-549, where only *CDK6* was downregulated upon FOXC1_KO (Figure 1F). To validate the FOXC1-induced gene expression changes in the CDK4-6/Rb/E2F pathway genes observed in RNA-seq data, clonogenic assays were performed to quantify changes in sensitivity to palbociclib, a CDK4/6 inhibitor that is used in treatment of ER+ tumors. Interestingly, only in Hs578t did the loss of FOXC1 further sensitize the cells to palbociclib, as seen by the reduction in the number of colony-forming units in the Hs578t_KO (Figure 1G). Both BT549_KO and MDA-MB-468_KO remained equally resistant to palbociclib as the parental cell lines and MDA-MB-231_KO showed no further sensitivity than the parental. FOXC1’s role in modulating the sensitivity of Hs578t to palbociclib, a clinically relevant CDK4/6 inhibitor, necessitates the study of FOXC1 gene targets in more detail while further emphasizing the diversity of FOXC1 function in TNBC.

### Genome-wide mapping of FOXC1 binding sites in four TNBC cell lines

To identify genes directly regulated by FOXC1, we mapped genome-wide FOXC1 binding sites using chromatin immunoprecipitation sequencing (ChIP-seq) in four TNBC cell lines. A high correlation was observed among the peaks identified in the three biological replicates within each cell line (Figure S3A), and subsequently, the three replicates were merged before peak calling. Nearly half of the FOXC1 binding sites were observed between promoters, 5′ UTR, and first intronic sequences, with about 25% present in distal intergenic, likely enhancer, regions (Figure S3B). This was supported by the average peak distribution relative to the transcription start site (TSS), with the majority of peaks at 10–100 kb from TSS (Figure S3C). In sum, 863 peaks were common between all four cell lines, with MDA-MB-468 containing the highest number of peaks (Figure S3D). There was higher conservation among FOXC1 peaks than FOXC1-associated DEGs across the four cell lines (863 common peaks versus 26 DEGs in Figure 1E) suggesting cell-line context dominates gene expression changes.

**Figure 2:**
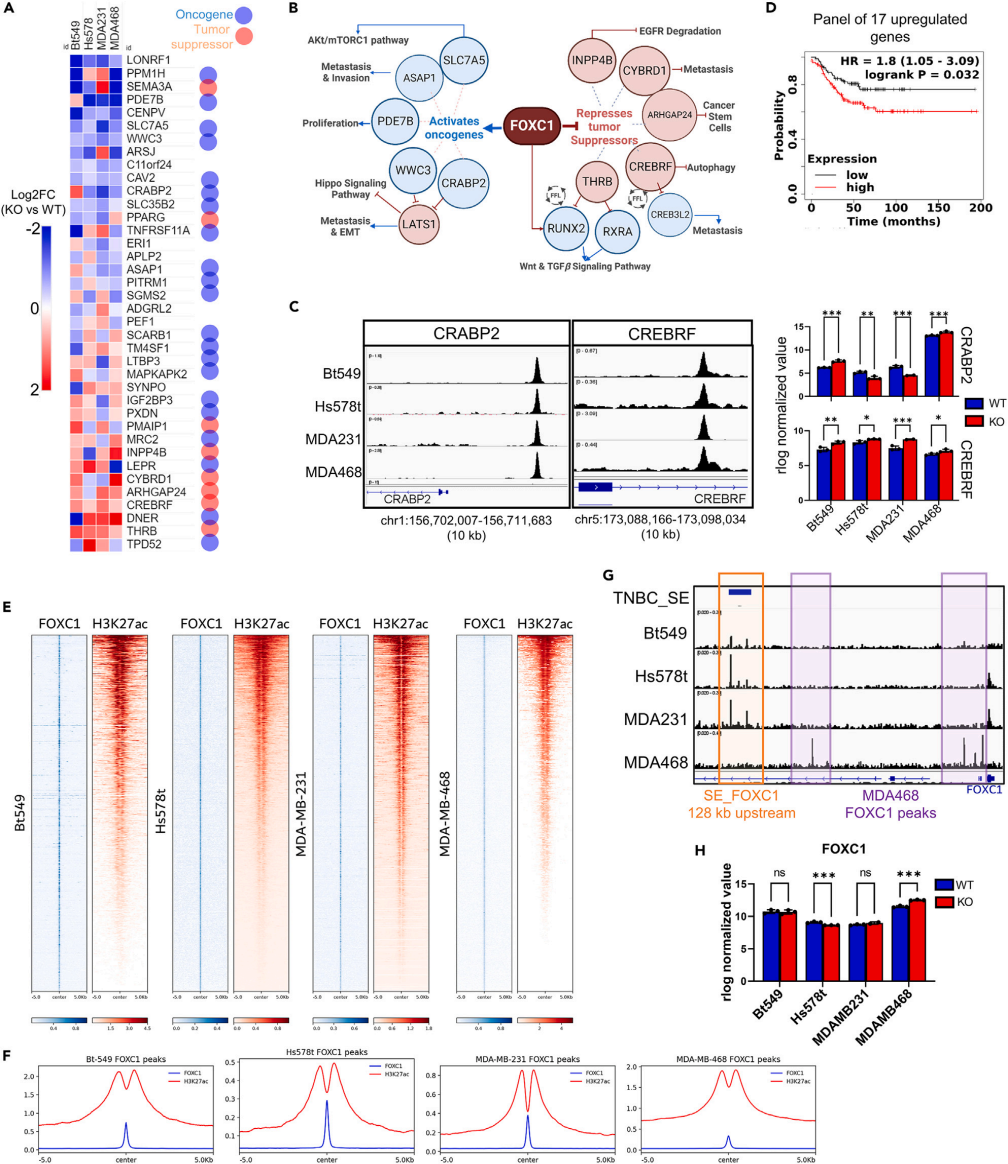
Genome-wide binding site analysis of FOXC1 in TNBC (A) Log2 (fold change) of normalized mRNA expression of 38 core, conserved gene targets that associate with a FOXC1 peak and are differentially regulated upon FOXC1 KO in all four TNBC cell lines. Dots represent literature-based evidence of the gene’s function, either as an oncogene (blue dots) or a tumor suppressor (red dots) in breast cancer. Nine genes (*THRB*, *CREBRF*, *SLC35B2*, *CAV2*, *C11orf24*, *WWC3*, *SLC7A5*, *CENPV*, and *LONRF1*) overlap with those in Figure 1E. (B) Schema illustrating the tumor suppressors and oncogenes directly regulated by FOXC1 and their respective role in tumorigenesis in TNBC. Created with BioRender. (C) ChIP-seq traces showing FOXC1 peaks near oncogene *CRABP2* and tumor-suppressor *CREBRF*. Bar plots depict normalized gene expression value in parental (WT, yellow) or FOXC1_KO (KO, blue) cell lines. Statistical significance determined using DESeq2 (∗*p*adj < 0.05, ∗∗*p*adj < 0.005, ∗∗∗*p*adj ≤ 0.0005). (D) Kaplan-Meier survival analysis of a panel of 17 core upregulated targets of FOXC1 of ER-(IHC), PR-(IHC) and HER2 negative (array) in 220 breast cancer patient samples from kmplot.com.^18^ (E) Heatmap of FOXC1 peaks associated with a H3K27ac signal in each of the four TNBC cell lines. (F) Averaged quantification of FOXC1 and H3K27ac signal intensity in heatmap in (D). (G) FOXC1 ChIP-seq peak traces near the super-enhancer 128 kb upstream of FOXC1 gene (orange box), and FOXC1 peaks unique to MDA-MB-468 (purple boxes). (H) Bar plots showing the normalized expression of FOXC1 mRNA in parental (blue, WT) and FOXC1_KO (red, KO) in TNBC cell lines. Statistical significance determined using DESeq2 (ns = not significant, ∗∗∗*p*adj < 0.0005).

In each cell line, we identified DEGs (FOXC1_KO vs. WT) within 50 kb of a peak and identified the functional, directly regulated gene targets of FOXC1. Thirty-eight genes were associated with peaks and significantly (*p*adj < 0.05) differentially expressed upon FOXC1 knockout in all four cell lines. We believe these stringently selected 38 genes are conserved FOXC1 direct targets in TNBC (Figure 2A). Within this list, FOXC1 represses expression of tumor suppressors (*CREBRF*, *THRB*, *INPP4B*, *ARHGAP24*, and *CYBRD1*) and activates the expression of oncogenes (*CRABP2*, *SLC7A5*, *PDE7B*, *ASAP1*, and *WWC3*) (illustrated in Figures 2B and 2C; Figure S4). The expression of the majority of these FOXC1 targets (21/38) is prognostic (*p* value < 0.05) in predicting relapse-free survival of patients with ER/PR/HER2 negative breast cancer (Figures S5A–S5D). The expression of a panel of 17 genes upregulated by FOXC1 correlates with poor survival (Figure 2D), underscoring the clinical relevance of FOXC1 regulated gene targets in TNBC. The expression of a panel of 12 downregulated genes did not significantly correlate with survival (Figure S5B).

The top *de novo* consensus binding motif for FOXC1 in high confidence peaks was similar in all four cell lines (Figure S3E). The consensus binding sites were overlaid with that identified from FOXC1-ChIP-seq binding data in an AML cell line (Fujioka cells)^19^ and revealed a slightly different consensus binding motif (Figure S3E), suggesting a degree of tissue/disease-specificity in FOXC1 binding sites (AML versus TNBC). TF motifs enriched within 100 bp of a FOXC1 peak using Homer revealed similarly enriched families in all four TNBC cell lines (Figure S3F). The highest enrichment was seen for 2 main families of motifs, the Forkhead/winged helix factor motif and the basic leucine zipper factor motif (bZIP), which includes TFs such as FOSL2, JUNB, and AP1. Enrichment of Kyoto Encyclopedia of Genes and Genomes (KEGG) pathways from among genes associated with FOXC1 peak in each cell line revealed Rap1, PI3-AKT, MAPK, Hippo, and Ras signaling pathways as conserved between all four cell lines, followed by TGFβ and Wnt signaling pathway as conserved in at least 2 cell lines (Figure S3G).

Using publicly available H3K27ac ChIP-seq data, we were able to observe that FOXC1 binding sites are present largely in active promoters and accessible enhancer regions, as these are enriched for H3K27ac histone modifications (Figure 2E). Quantification of the FOXC1 and H3K27ac signal intensity at each overlapped site revealed that MDA-MB-468 had the smallest fraction of FOXC1 peaks associated with H3K27ac signal (Figure 2F). Thus, although MDA-MB-468 had the highest number of FOXC1 peaks (Figure S3D), most peaks did not associate with active chromatin regions. In contrast, in MDA-MB-231, which had the smallest number of FOXC1 peaks, a majority associated with H3K27ac histone signal, suggesting that FOXC1 has a more “active” role in that cell line.

Super-enhancers (SEs) are large clusters of transcriptionally active enhancers that drive oncogene expression.^20^^,^^21^ In order to identify TNBC-critical SEs that are bound by FOXC1, we overlapped our ChIP-seq data with the publicly available list of 331 TNBC-specific SEs that are near genes upregulated in TNBC.^20^^,^^21^ In total, FOXC1 peaks were observed at fifteen TNBC specific SEs in all four TNBC cell lines (Figure S3H). However, MDA-MB-468 has been suggested to have an epigenetic landscape that is similar to that of non-TNBC breast cancer cell lines and lacks many TNBC-specific SEs.^20^ Thus, excluding MDA-MB-468, 19 SEs were common between Bt-549, Hs578t, and MDA-MB-231 and include important SEs, such as those associated with oncogenes *IL6*, *CXCL8*, *FOXC1*, and *CDK6*. Although not a comprehensive analysis of all SEs associated with FOXC1 peaks, these 19 TNBC-specific SEs highlight FOXC1’s role in regulating the respective key target genes that control the TNBC phenotype.

An SE 128 kb upstream of the *FOXC1* gene that has been shown to activate the expression of FOXC1 was absent in MDA-MB-468 (Figure 2G), supporting a previous study.^20^ Further, FOXC1 seems to repress its own gene expression in MDA-MB-468, as the normalized FOXC1 mRNA levels increase 2-fold in the absence of FOXC1 protein (Figure 2H). A possible repressive FOXC1 binding site is visible upstream of the *FOXC1* gene in MDA-MB-468 and not in the other cell lines (Figure 2G), which could indicate the presence of negative feedback of FOXC1 mRNA in MDA-MB-468. Moreover, only in MDA-MB-468 does FOXC1 directly bind and repress expression of receptor *ERBB2* (HER2), *EGFR*, and *FOXA1*, genes important for hormone/growth signaling (Figure S6). EGFR expression, in particular, decreases 2-fold upon loss of FOXC1, suggesting that FOXC1 drives proliferation in MDA-MB-468 via the upregulation of EGFR expression. MDA-MB-468 is the only basal-like/basal A TNBC subtype in this analysis, and together with our observation that MDA-MB-468 has a distinct gene expression (Figures S2A and S2B) and genome-wide binding pattern, the data suggests that FOXC1’s function in MDA-MB-468 is unique and that the cell line has characteristics atypical of TNBC and reminiscent of luminal subtypes. Cumulatively, the diversity of FOXC1 function in TNBC, likely due to the miscellaneous nature of TNBC classification, necessitates identification of the core, conserved gene targets that can inform selection of appropriate targeted therapies.

### Identifying core, conserved FOXC1 gene targets in TNBC

**Figure 3:**
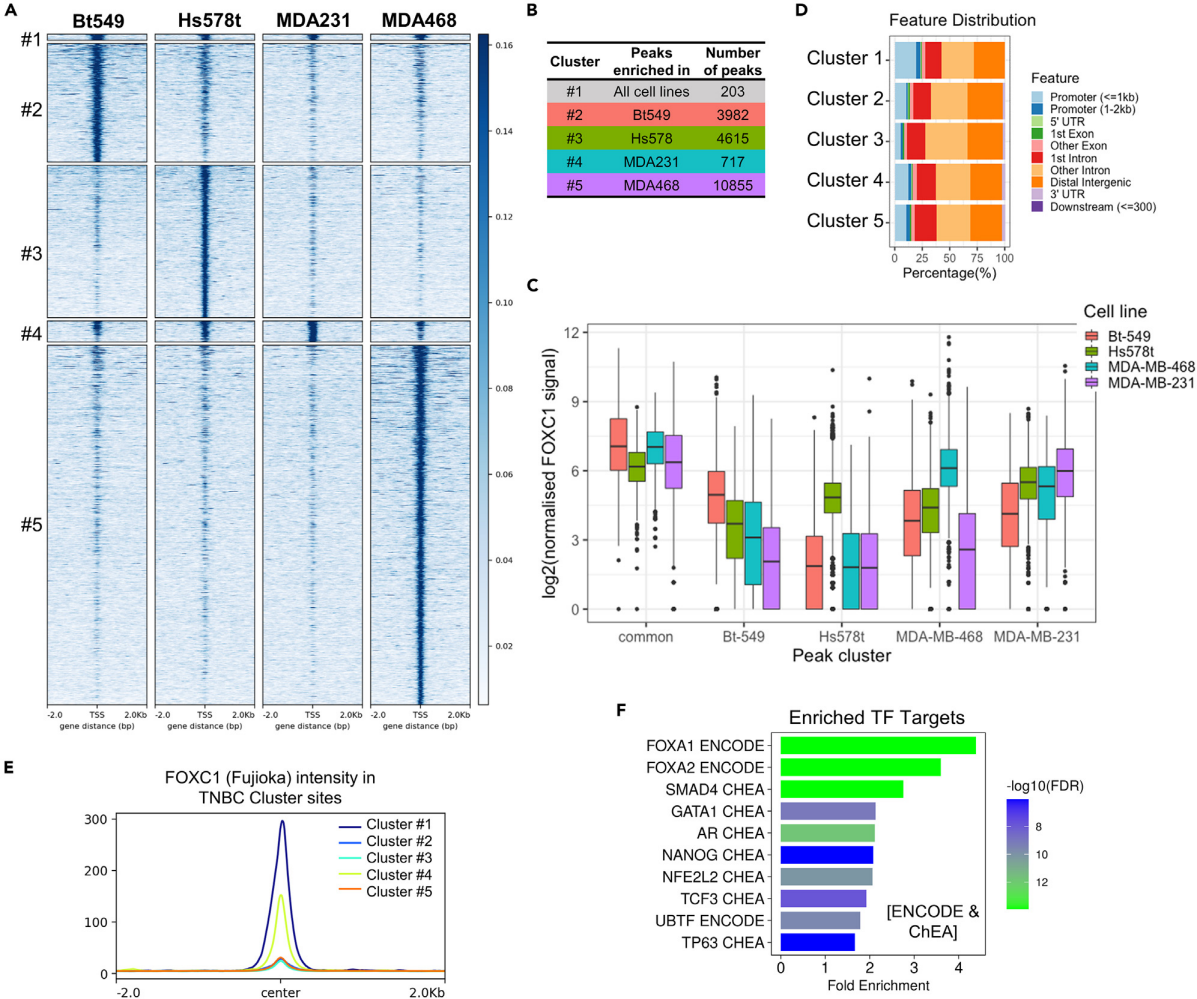
Core, conserved FOXC1 gene targets in TNBC (A) Unsupervised k-means clustering of peaks among the four TNBC cell lines into 5 clusters: cluster 1, peaks in all cell lines; clusters 2–5, peaks dominant in a particular cell line. (B) Number of peaks in each cluster identified in (A). (C) Quantification of the FOXC1 signal in each of the five clusters. One-way ANOVA followed by Tukey’s post-hoc test, comparing cell lines within each peak cluster. (D) Location of FOXC1 peaks near genome features in each of the five clusters. (E) The average intensity of FOXC1 peaks in Fujioka (AML) cell lines overlaid onto the five clusters of FOXC1 binding sites in TNBC from (A). (F) Top enriched transcription factors that have similar gene targets as core targets of FOXC1, using the TF target (ENCODE and ChEA consensus from ChIP-X experiments), generated using ShinyGO.^22^

Unsupervised k-means clustering was used to group FOXC1-binding peaks, leading to the classification of peaks into five clusters with cluster 1 (binding sites present in all four cell lines) followed by four cell-line dominant clusters, where FOXC1 peak intensity was dominant in that respective cell line (Table S2) (Figure 3A). The cluster analysis indicates that the majority of FOXC1 binding events are largely cell-line specific since cluster 1 was the smallest cluster (203 peaks) (Figure 3B). Nonetheless, cluster 1 contained peaks with overall the highest intensity, as seen by the quantification of the normalized FOXC1 signal in each cluster (Figure 3C), indicating that the strongest FOXC1 binding sites are conserved across all four cell lines. FOXC1 peak intensity was also high within a respective cell line’s dominant cluster, except in the case of MDA-MB-231, where all cell lines displayed equal FOXC1 intensity at peaks (Figure 3C). This suggests that the MDA-MB-231 cluster is least exclusive, i.e., MDA-MB-231 FOXC1 peaks are most likely to be present in the other 3 cell lines as well. Cluster 5, consisting of FOXC1 peaks that were enriched in MDA-MB-468, was the largest (∼10,000 peaks) and most unique cluster. Furthermore, core FOXC1 peaks in cluster 1 were over-represented at promoter features when compared to the other clusters (Figure 3D), suggesting that conserved FOXC1 binding sites are more likely to be found at promoters and cell-line specific FOXC1 binding sites are likely at enhancer regions. Lastly, these results suggest that, even in cell lines where FOXC1 is only moderately expressed, such as MDA-MB-231, the core peaks remain conserved. High-FOXC1 expressing cell lines did not possess an additional cluster of unique FOXC1 peaks (such as a cluster of peaks present in only Hs578t and Bt549), but rather possessed individual cell-line specific clusters.

We compared our FOXC1 binding profile in TNBC to that published in an AML cell line model (Fujioka cell line).^19^ FOXC1 peak intensity in Fujioka (AML) cell lines was grouped along the five TNBC clusters in Figure 3A and revealed highest intensity over cluster 1 binding sites, followed by those of the MDA-MB-231 dominant cluster 4 (Figures 3E and S7). In summary, the conserved, highest-intensity FOXC1 binding sites in TNBC are also bound by FOXC1 in AML. Genes regulated by FOXC1 binding sites at cluster 1 and cluster 4 peaks could thus represent the core gene targets of FOXC1 across disease types.

Annotation of peaks with genes present within 50 kb in cluster 1 or cluster 4 yielded a list of 1,646 genes. To identify other TFs that bind upstream of the same genes, we performed enrichment analysis using the TF Target database (consensus of ChIP-seq experiments in ENCODE and ChEA). The highest fold enrichment, nearly 4-fold, was observed for FOXA1 targets (Figure 3F), followed by FOXA2 and SMAD4. FOXA1 is a pioneer TF that establishes the ERα transcriptional complex along with other cofactors, such as GATA3 and NR2F2.^6^^,^^23^ Shared gene targets between FOXC1 and FOXA1 suggests that FOXC1 could regulate many crucial ERα targets in TNBC and motivated us to identify possible co-factors of FOXC1.

### FOXC1 interacts with nuclear receptor NR2F2

**Figure 4:**
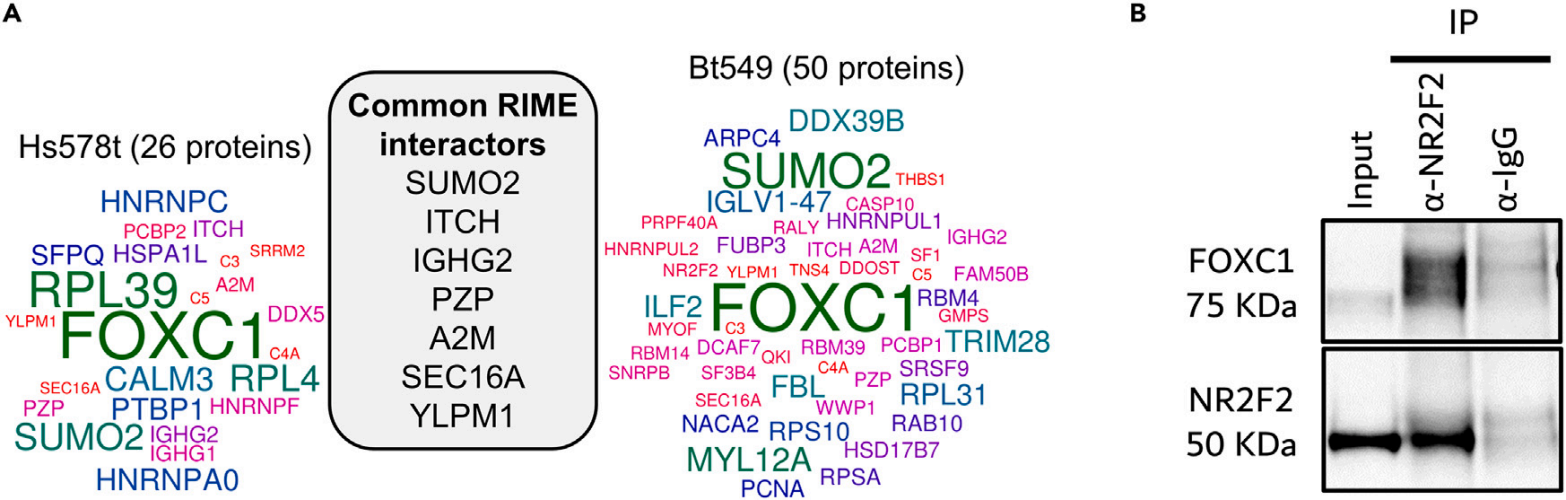
Identification of FOXC1 cofactors (A) Protein co-interactors of FOXC1 identified in Hs578t and BT-549 via RIME in two biological replicates, after subtracting those identified in negative IgG control. (B) Western blot depicting co-immunoprecipitation of FOXC1 using antibody against NR2F2 with input and IgG negative controls.

To identify protein cofactors of FOXC1 in TNBC, we immunoprecipitated proteins crosslinked with FOXC1 and analyzed interacting partners via mass spectrometry (rapid immunoprecipitation mass spectrometry of endogenous proteins [RIME]),^24^ in BT-549 and Hs578t, as these two cell lines have higher FOXC1 protein levels. Fifty and 26 unique proteins were identified to interact with FOXC1 in BT-549 and Hs578t, respectively, in two biological replicates, after filtering for non-specific proteins that were immunoprecipitated using an IgG negative control (Figure 4A; Table S3).

Ten proteins were common between the two cell lines, excluding FOXC1, which was the most significantly enriched protein with 12 unique peptides, validating the specificity of the antibody used and the method for isolating associated proteins. The strongest interactor was SUMO2, a small ubiquitin-like modifier, which has been shown to SUMOlyate FOXC1 and regulate its activity.^25^ Other high-confidence co-factors identified include TRIM28, which interacts with FOXC1 at the promoter of the Wnt5A gene,^26^ and ITCH, an E3 ubiquitin ligase.

Interestingly, NR2F2, a nuclear receptor involved in the ERα transcriptional complex, was identified as one of the interacting partners of FOXC1 in BT-549, albeit with low peptide coverage. This prompted us to test whether FOXC1 and NR2F2 interact. We overexpressed FOXC1 and NR2F2 in BT-549, co-immunoprecipitated proteins bound to NR2F2 using anti-NR2F2, and assayed for the presence of FOXC1. FOXC1 was enriched specifically in the NR2F2 immunoprecipitated reaction above that of the IgG negative control (Figure 4B), suggesting that the two proteins interact and thus could cooperatively function to regulate gene expression in TNBC.

### FOXC1 and FOXA1/NR2F2 bind similar sites in respective breast cancer subtypes

**Figure 5:**
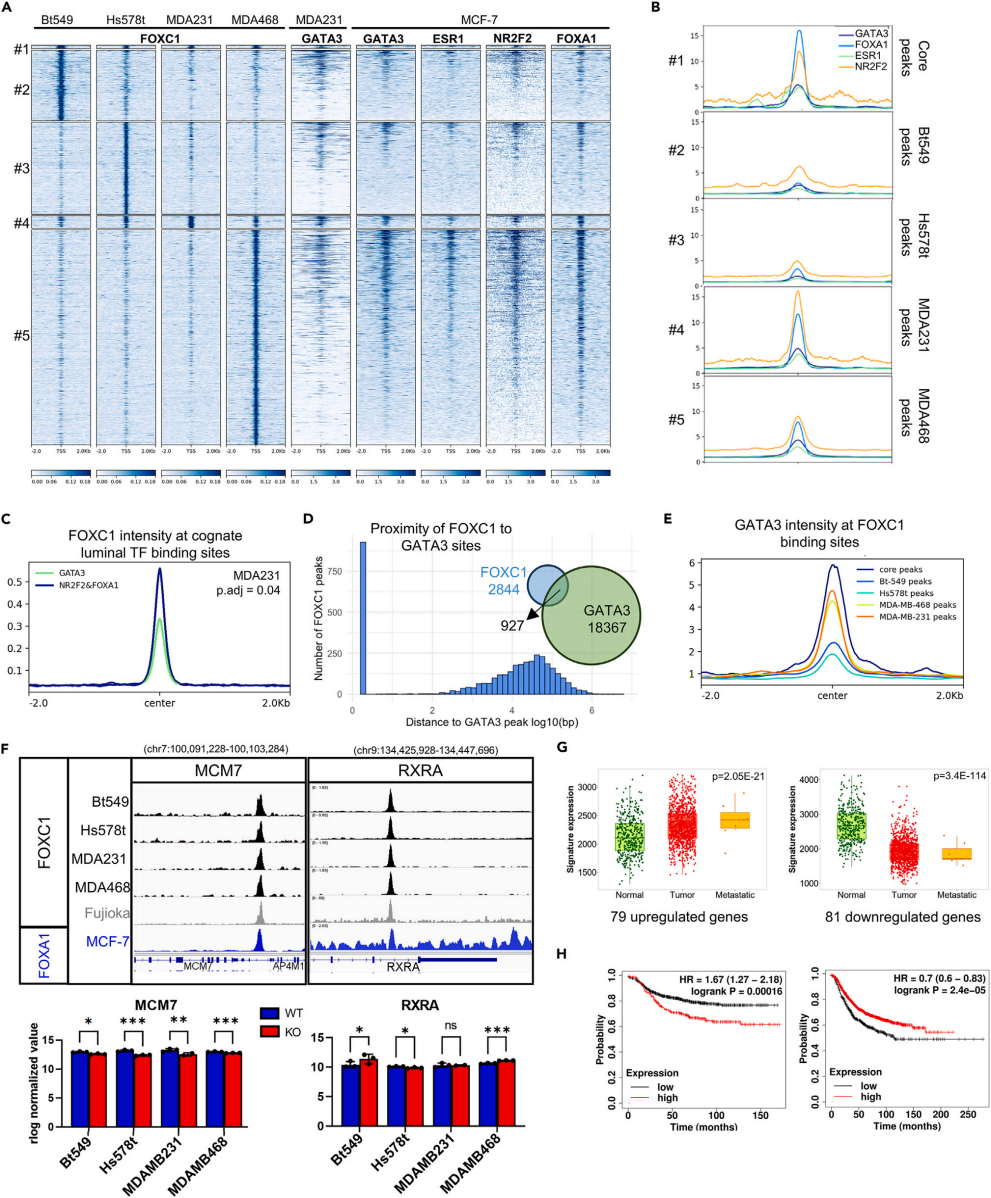
Core FOXC1 binding sites in TNBC are bound by FOXA1/NR2F2 in luminal breast cancer (A) Heatmaps depicting overlap of ChIP-seq data of transcription factors (GATA3, ESR1, NR2F2, and FOXA1) from MCF-7 luminal breast cancer cell lines with the five TNBC clusters of FOXC1 peaks identified in Figure 3A. (B) Average ChIP-seq tag intensity of transcription factors (GATA3, ESR1, NR2F2, and FOXA1) in MCF-7 over TNBC peak clusters from previous panel. (C) Average intensity of FOXC1 peaks in MDA-MB-231 at sites that are bound by FOXA1+NR2F2 versus GATA3 (cognate sites) in MCF-7. Adjusted *p* value calculated using a Tukey post-hoc test. (D) Proximity of FOXC1 to GATA3 binding sites in MDA-MB-231 and the number of peaks overlapping between FOXC1 and over-expressed GATA3 in MDA-MB-231. (E) Average intensity of GATA3 over FOXC1 peaks in each of the five clusters in Figure 3A. (F) ChIP-seq traces (above) showing FOXC1 peaks in TNBC and AML cell lines and FOXA1 peaks in MCF-7 near MCM7 and RXRA genes, two critical genes in breast cancer and barplots (below) show changes in normalized gene expression from RNA-seq of the genes in WT (blue) or FOXC1_KO (red). Statistical significance determined using DESeq2 (ns = not significant, ∗*p*adj < 0.05, ∗∗∗∗*p*adj < 0.00005). (G) Change in mean expression of up- or downregulated core genes in all breast invasive carcinomas based on RNA-seq data from the GEO database in tumor, adjacent normal or metastatic samples (TNMplot.com^29^). *p* value shown calculated using Kruskal-Wallis tests. (H) Kaplan-Meier survival analysis of upregulated (left) and downregulated (right) core gene targets of FOXC1 on 2032 breast cancer patient samples from kmplot.com.^18^

In luminal breast cancer, ERα is the major driver of estrogen-responsive proliferation.^27^ Inaccessible chromatin is first bound by the pioneer factor FOXA1, which then recruits chromatin modifiers to open up the chromatin. ERα is recruited onto a subset of FOXA1 sites, which are also bound by GATA3 and/or NR2F2. Approximately 85% of ERα bound sites are bound by FOXA1, GATA3, and NR2F2 as part of the ERα transcriptional complex, and as such, these TFs share many of the binding sites.^24^ We analyzed whether FOXC1 binds and regulates similar targets in TNBC as those regulated by the ERα-associated TFs in luminal breast cancer. ESR1 (ERα), FOXA1, and GATA3 are not expressed in TNBC, and NR2F2 is variably expressed, as seen by mRNA expression data and normalized protein expression (Figure S8A^28^). Thus, we utilized publicly available ChIP-seq data for ESR1, FOXA1, GATA3, or NR2F2 generated in luminal MCF-7 cell line and clustered the respective binding sites to our FOXC1 ChIP-seq data (Figure 5A). Some overlap was observed between FOXC1 peaks in TNBC and the four TFs in MCF-7, with highest overlap in cluster 1, consisting of core FOXC1 peaks that were common across all four TNBC cell lines. Peak intensity of each TF in the respective five clusters was quantified (Figure 5B). While the intensity of FOXA1, NR2F2, GATA3, and ESR1 remained relatively equal in the Bt-549 and Hs578t clusters 2 and 3, respectively, intensity of FOXA1 and NR2F2 was significantly higher in clusters 1 and 4 when compared to either ESR1 or GATA3 (Figure 5B). This suggests that FOXA1 and NR2F2 preferentially bind at the identified “core peaks” of FOXC1, represented by clusters 1 and 4, in the luminal cell line they are expressed in.

Next, we did the reverse analysis and quantified FOXC1 intensity to binding sites assigned a FOXA1, GATA3, or NR2F2 identity depending on whether that site was bound by the TFs in MCF-7 (“cognate sites”). Indeed, intensity of FOXC1 peaks in MDA-MB-231 was observed to be significantly higher in sites bound by FOXA1+NR2F2 in MCF-7 versus those bound by GATA3 (Figure 5C). Since cluster 1 + cluster 4 peaks in Figure 5A account for high-intensity FOXC1 binding sites in MDA-MB-231, this result independently shows that FOXC1 preferentially binds at sites that are bound by FOXA1 and NR2F2 in MCF-7. In the other cell lines as well, similar patterns of FOXC1 binding preferences emerged, where FOXC1 binding intensity was significantly higher at cognate sites that were bound by FOXA1, NR2F2, or a combination of all TFs in luminal MCF-7 when compared to sites bound by GATA3 alone (Figure S8B).

The aforementioned analysis compared FOXC1 ChIP-seq data in TNBC cell lines to that of other TFs in luminal MCF-7 cell line. To validate whether exogenous expression of a luminal TF would lead to co-binding at sites bound by FOXC1, we compared our FOXC1 ChIP-seq data from MDA-MB-231 with publicly available ChIP-seq data in MDA-MB-231 with exogenous expression of GATA3.^30^ About 25% of FOXC1 in MDA-MB-231 were bound by GATA3 as well, and most GATA3 peaks within 50 kb of a FOXC1 peak were present within 100 bp of the FOXC1 peak (Figure 5D), suggesting that the two proteins can bind to similar binding sites, at least in MDA-MB-231. Of the 445 DEGs in MDA-MB-231 that were associated with FOXC1 peaks, a GATA3 peak was seen at 145 genes, suggesting co-regulation (Figure S8C). The average intensity of GATA3 was equally intense in cluster 1 FOXC1 peaks and cluster 4, the MDA-MB-231 cluster (Figure 5E), again emphasizing the importance of the identified core FOXC1 peaks. However, our data also suggest that FOXC1 represses expression of GATA3 via a binding site in the first intron, in all cell lines except MDA-MB-231, where an increase in GATA3 expression was observed (Figures S8D and S8E). GATA3 has been previously shown to repress FOXC1 expression,^17^^,^^27^ suggesting that the two proteins are mutually exclusively expressed, and that exogenous expression as in the aforementioned case of MDA-MB-231 ChIP-seq data could result in abnormal function.

In summary, along with the observation that FOXC1 and NR2F2 co-interact, it is likely that core binding sites of FOXC1 in TNBC identified here and represented by cluster 1 and cluster 4 peaks, are sites where FOXA1/NR2F2 are bound in luminal breast cancer (and vice versa). To identify genes functionally regulated by the identified peaks, we assembled a list of genes within 50 kb of a peak in cluster 1 and cluster 4 that were differentially expressed upon FOXC1 knockout in at least 3 cell lines. This yielded an exhaustive list of 164 genes that we termed as the “core targets of FOXC1” (Figure S9A; Table S4). Pathway enrichment using Hallmark gene sets (MSigDB) revealed enrichment of key cancer hallmarks such as epithelial-to-mesenchymal transition (EMT), IL-2 STAT5 signaling, and early response to estrogen (Figure S9B). Among the genes are key prognostic TNBC-specific biomarkers, drug-targetable receptors, kinases, lncRNA, and crucial TFs (Figures 5F, S9C, and S9D) as well as novel ligands that were previously unknown to be under direct regulation by FOXC1 (Figure S10).

Clinical relevance for the previously identified core targets was evaluated by visualizing mean expression of either the upregulated or downregulated genes in normal, tumor, or metastasized patient samples (TNMplot.com^29^). As expected, the panel of 81 genes repressed by FOXC1 was seen to have significantly lower expression in tumor and metastatic breast invasive carcinoma samples, inclusive of all subtypes (Figure 5G). Likewise, mean expression of 79 upregulated genes was greater in tumor compared to normal samples. Moreover, the above up- and downregulated gene panels were seen to have significant prognostic value in breast cancer patient samples, regardless of subtype (Figure 5H). High expression of genes activated by FOXC1 was seen to correlate with poor survival whereas high expression of genes repressed by FOXC1 correlated with better survival (Kaplan-Meier Plotter^18^). Together, the correlation of FOXC1 regulation with the mean expression and prognostic value of the up- and downregulated targets in all breast invasive carcinomas, irrespective of subtype, indicates that the identified 164 core FOXC1-regulated genes are important targets of transcriptional regulation in breast cancer.

## Discussion

In this study, we first establish that, although phenotypically FOXC1 affects the major tumour-associated properties in TNBC, only 26 genes are differentially expressed upon loss of FOXC1 across four TNBC cell lines with varying levels of FOXC1 expression. The lack of conserved DEGs in RNA-seq suggests cell-line specific and context dependent function for FOXC1 in TNBC. The heterogeneity of FOXC1 function in TNBC is reflective of the heterogeneity of TNBC and emphasizes the obstacles in identifying unified targeted therapies. Given that FOXC1 is over-expressed in many cancers^10^ and given its role as a clinically useful biomarker and potential therapeutic target, identification of core target genes regulated by FOXC1 and its co-factors sheds light on its function and elucidates mechanisms of tumor progression.

By analyzing genome-wide FOXC1 binding sites in four cell lines, we identify conserved gene targets of FOXC1 in TNBC and we show that these core targets are critical for TNBC pathogenesis (Figure 2A) and have clinical relevance (Figure 2D). Within the conserved TNBC regulon, we show that FOXC1 represses expression of the tumor suppressors *CREBRF*, *THRB*, *ARHGAP24*, and *INPP4B* and activates expression of the oncogenes *CRABP2*, *WWC3*, *SLC7A5*, *ASAP1*, *PDE7B*, and *IGF2BP3* (illustrated in schematic Figures 2B and S4). To illustrate an example, the FOXC1 target CREBRF is a tumor suppressor that regulates autophagy.^31^ CREBRF facilitates the degradation of CREB3 family TFs, which promote metastasis.^32^ In our data, FOXC1 directly regulates both CREBRF and the downstream CREB3-family TF, CREB3L2, resulting in an apparent feedforward regulatory loop (Figure 2B). Likewise, an oncogene directly upregulated by FOXC1 is the amino acid transporter SLC7A5 (LAT1), which mediates uptake of leucine in exchange for glutamine, and is typically over-expressed in TNBC, with higher expression correlating with worse prognosis.^33^ FOXC1 activates expression of SLC7A5 nearly 2-fold in all four cell lines via binding site within the first intron (Figure S4D). As leucine is a regulator of mTORC1, SLC7A5 has been speculated to promote proliferation via the Akt/mTORC1 pathway and is a target in preclinical trials.^34^ Lastly, many ligands and proteins regulated by FOXC1 such as DDIT4, LONRF1, CHI3L2, and SYNPO (Figure S10) pose as attractive targets for further functional studies in TNBC, that although have no studied role in TNBC, their prognostic value and function in other malignancies suggests an important function relevant to TNBC.

Among the co-factors of FOXC1 identified in this study are SUMO and TRIM28, which have been shown to previously interact with FOXC1,^25^^,^^26^ as well as two newly identified protein interactors, the E3 ubiquitin ligase ITCH and nuclear receptor NR2F2. FOXC1 protein levels are regulated by post-translational modifications via the ubiquitin 26S proteasomal degradation pathway,^35^ suggesting that ITCH could be the E3 ligase responsible for ubiquitinating FOXC1. Targeting E3 ubiquitin enzymes for cancer therapy has long been considered an attractive mechanism-based drug discovery approach and the possibility of regulating FOXC1 activity via targeting ITCH remains to be seen.^36^

The interaction between FOXC1 and NR2F2 was confirmed using co-immunoprecipitation. NR2F2 is an orphan nuclear receptor in the steroid hormone receptor family that influences cell invasion, migration and EMT in breast cancer.^37^ Among breast cancer subtypes, its expression is highest in the luminal A subtype; although in the TNBC cell lines Bt549 and MDA-MB-231 used in this study, NR2F2 expression is comparable to that in MCF-7, a luminal A cell line (Figure S8A). In MCF-7, NR2F2 has been shown to be present in the ERα transcriptional complex along with FOXA1 and GATA3, where it influences ERα binding on genes driving proliferation and metastasis.^23^ A small molecule inhibitor of NR2F2 was recently shown to reduce prostate cancer tumor growth, specifically by interrupting the interaction of NR2F2 with FOXA1, a master regulator in prostate tumorigenesis.^38^ Whether NR2F2 and FOXC1 influence their respective binding in TNBC remains to be determined, but the possibility of using the NR2F2-inhibitor to disrupt the interaction with FOXC1 in TNBC presents a potential targeted-therapy approach for basal-like breast cancer.

By analyzing genome-wide binding across TNBC cell lines with varying FOXC1 protein expression, we were able to identify the core set of conserved FOXC1 peaks. Intriguingly, we found that high-FOXC1 expressing cell lines did not have a common set of peaks, suggesting that apart from the core sites identified in this study, FOXC1 binding sites are largely cell-line specific. Next, using publicly available binding site data of ERα-associated TFs, we show that the identified core sites bound by FOXC1 in TNBC are bound by FOXA1 and NR2F2 in ER+ breast cancer. FOXA1 is a pioneer factor that directs ERα binding in ER+ breast cancer^39^ and controls expression of critical genes. Knockdown of FOXA1 in ER+ breast cancer reduces luminal-lineage proliferation.^40^^,^^41^ That many FOXA1 targets are regulated by core FOXC1 binding sites in TNBC emphasizes the importance of these targets in TNBC and highlights the need for further functional studies. The 164 core targets of FOXC1 includes many important ligands, receptors, kinases, lncRNA, and TFs such as *MCM7*, *RXRA*, *CDK6*, *RUNX2*, *NEAT1*, *and ABL1* (Figure S9). To highlight, FOXC1 was seen to regulate expression of *RXRA* that encodes retinoid X receptor alpha (Figure 5F). RXRA in turn forms heterodimers with TRβ, encoded by THRB, which is strongly repressed by FOXC1 as well (Figures 2B and S4A). THRB is a known tumor suppressor in TNBC, whose high expression correlates with better survival.^35^ Upon binding the agonist T3, TRβ form heterodimers with RXR receptors and promotes tumor suppressive pathways.^36^ Interestingly, RXR motifs are enriched near FOXA1 peaks, but only in an ESR1-mutated, endocrine resistant background.^42^ Moreover, the same study reported stimulation of RXR:TRβ activity using T3 in the context of breast cancer. TRβ inhibits the expression of RUNX2, an oncogene in TNBC,^37^ whose expression is in turn upregulated by FOXC1, in another example of a coherent feedforward loop (Figures 2B and S9D). Lastly, both FOXC1 and FOXA1 have multiple binding sites within the CDK6 gene that overlaps with a TNBC-specific SE (Figure S9D). CDK6 is a clinically important target, since CDK4/6 inhibitors are used in treatment of ER+ breast cancer, and clinical trials are currently evaluating their effectiveness in TNBC.^43^ Here we show that FOXC1 drives sensitivity to palbociclib, a CDK4/6 inhibitor in Hs578t, by regulating key genes in the cyclinD1/CDK4/6 Rb1/pathway such as *CDK6*, *E2F7*, *MCM7*, and *HMGB2* (Figures 1F and 1G). Nonetheless, many TNBC cell lines, except those of the luminal androgen receptor (LAR) subtype, are resistant to CDK4/6 inhibitors via mechanisms not clearly defined but involving CDK2 and/or AR expression, and p53, PTEN, or Rb1 status.^44^^,^^45^^,^^46^

We have delineated the core, conserved function of FOXC1 in TNBC, and show that these core sites are parallelly regulated by FOXA1 in ER+ breast cancer. The core gene targets of FOXC1 identified in this study form a conserved list of genes important in breast cancer, a molecular signature for FOXC1-overexpressing cancers and could present as attractive targets for subtype-independent breast cancer therapies. To our knowledge, this is the first global genomic analysis of FOXC1’s molecular role in TNBC, and broadens considerably the known regulon of FOXC1, a prognostic marker and crucial TF that is over-expressed in TNBC/basal-like tumors.^10^

### Limitations of the study

A crucial facet of TNBC is its heterogeneous nature, which leads to selective growth of resistant-sub populations upon drug treatment.^2^ Cell line models used in this study do not fully encapsulate the heterogeneity of TNBC, but rather represent the heterogeneity in FOXC1 expression. The small number of core conserved FOXC1 targets identified in the transcriptomic analysis could be due to the inherent noise in RNA-seq data, considering the minimal three replicates performed in this study that afford very narrow margins for significance. Another limitation of this study is the use of single FOXC1_KO CRISPR clones from each cell line for the RNA-seq analysis, which is compensated by the fact that the CRISPR KO from the four cell lines were grouped and used for comparative analysis and generation of core gene target lists, in effect functioning as four independent CRISPR clones. ChIP-seq datasets reproducibility is impeded by cell line variation, antibodies, or downstream analysis, and our study achieves robustness by using three biological replicates from four different TNBC cell lines. Future work will focus on validating the core targets and the biological processes these targets are involved in, as well as expanding FOXC1’s role to patient tumor samples that are more complex and heterogeneous compared to cell lines using single-cell omics. In addition, therapies that potentially target FOXC1 via modulating its interaction with cofactors can be investigated, as discussed in the case of NR2F2 and ITCH previously. Overall, this rigorous study adds to the available public data on transcription factors binding data, allowing for comprehensive and global analysis in the future, that can take into account the network of transcription factors that define cell fate and control oncogenesis.

## STAR★Methods

### Key resources table

**Table:** 

REAGENT or RESOURCE	SOURCE	IDENTIFIER
**Antibodies**		

FOXC1 (ChIP-seq and RIME)	Simeoni et al.^19^	
Recombinant Anti-FOXC1 antibody [EPR20685] (For WB, Co-IP)	Abcam	ab227977; RRID:AB_2916124
Mouse Anti-Actin, beta Monoclonal Antibody, Unconjugated	Abcam	ab6276; RRID:AB_2223210
Goat Anti-Rabbit IgG - H&L Polyclonal antibody, Hrp Conjugated	Abcam	ab6721; RRID:AB_955447
Anti-NR2F2 (ARP-1 (B-2)) (Co-IP)	Santa Cruz Biotech	sc-271265X; RRID:AB_10608838
Recombinant Anti-NR2F2 antibody [EPR18443] (WB)	Abcam	ab240387; RRID:AB_2895604
Chicken polyclonal Secondary Antibody to Mouse IgG - H&L	Abcam	ab6706; RRID:AB_956003

**Chemicals, peptides, and recombinant proteins**		

DSG (disuccinimidyl glutarate)	Thermoscientific	Catalog number: 20593, 50MG
PMSF (Phenylmethylsulfonyl fluoride)	Roche	Catalog number: 10837091001
TrueCut™ Cas9 Protein v2	Invitrogen	Catalog number: A36499
Lipofectamine CRISPRmax	ThermoFisher	Catalog number: CMAX00003
Propidium Iodide	Thermoscientific	Catalog number: P3566
GelTRex LDEV	Thermoscientific	Catalog number: A1413201

**Critical commercial assays**		

GeneArt™ Genomic Cleavage Detection Kit	Invitrogen	Catalog number: A24372
Guide-it™ Genotype Confirmation Kit	Takara	Catalog number: 632611
Pierce™ BCA Protein Assay Kit	ThermoFisher Scientific	Catalog number: 23225
RNeasy Mini Kit	Qiagen	Catalog number: 74106
RNase-Free DNase Set	Qiagen	Catalog number: 79254
Illumina stranded mRNA library prep kit	Illumina	Catalog number: 20040534
TruSeq Stranded mRNA Library Prep Kit	Illumina	Catalog number: 20020594
Qubit BR RNA kit	Invitrogen	Catalog number: Q10211
Qubit dsDNA BR kit	Invitrogen	Catalog number: Q32850
KAPA quantification kit	Roche	Catalog number: 07960140001

**Deposited data**		

Raw and processed ChIP-seq and RNA-seq sequencing data generated in this study	NCBI’s Gene Expression Omnibus https://www.ncbi.nlm.nih.gov/geo/	GEO: GSE213841
Mass spectrometry proteomics data generated in this study	ProteomeXchange Consortium via the PRIDE partner repository	PRIDE: PXD053261
Raw data from Figure 1	Mendeley Data	Mendeley Data: https://doi.org/10.17632/b4c28szwyn.2
Publicly available dataset for GATA3 ChIP-seq in MDA-MB-231	NCBI’s Gene Expression Omnibus	GEO: GSE162003
Publicly available dataset for ESR1 ChIP-seq in MCF-7	NCBI’s Gene Expression Omnibus	GEO: GSE170139
Publicly available dataset for FOXA1 ChIP-seq in MCF-7	NCBI’s Gene Expression Omnibus	GEO: GSE105305
Publicly available dataset for GATA3 ChIP-seq in MCF-7	NCBI’s Gene Expression Omnibus	GEO: GSE127656
Publicly available dataset for NR2F2 ChIP-seq in MCF-7	NCBI’s Gene Expression Omnibus	GEO: GSM1010837
Publicly available dataset for H3K27ac ChIP-seq in BT549 & MDA-MB-468	NCBI’s Gene Expression Omnibus	GEO: GSE69107
Publicly available dataset for H3K27ac ChIP-seq in Hs578t.	European Nucleotide Archive, https://www.ebi.ac.uk/ena/browser/home	ENA: PRJEB33558
Publicly available dataset for H3K27ac ChIP-seq in MDA-MB-231	NCBI’s Gene Expression Omnibus	GEO: GSE38548

**Experimental models: cell lines**		

MDA-MB-231	AddexBio	C0006002, RRID:CVCL_0062
MDA-MB-468	AddexBio	C000600, RRID:CVCL_0419
Bt549	AddexBio	C0006017, RRID:CVCL_1092
Hs578t	AddexBio	C0006016, RRID:CVCL_0332
MDA-MB-231_KO	Generated in this study	This manuscript
MDA-MB-468_KO	Generated in this study	This manuscript
Bt549_KO	Generated in this study	This manuscript
Hs578t_KO	Generated in this study	This manuscript

**Oligonucleotides**		

Forward PCR Primer for FOXC1 (RR042)	5′CCATGAGCGTGTACTCGCA	This manuscript
Reverse PCR Primer for FOXC1 (RR043)	5′GCGCATCCAGGACATCAAGA	This manuscript
Synthetic guide RNA for FOXC1 knockout (CRISPR684819_SGM)	GAACGCCCCGGACAAGAAGA	Synthego, ThermoFisher Scientific
Synthetic guide RNA for FOXC1 knockout (CRISPR684834_SGM)	GACAAGAAGATCACCCTGAA	Synthego, ThermoFisher Scientific

**Software and algorithms**		

ImageJ	https://imagej.nih.gov/ij/	RRID: SCR_003070https://imagej.nih.gov/ij/
FlowJo 10 v10.6.1	Becton Dickinson	RRID: SCR_008520https://www.flowjo.com
GraphPad Prism v8.3.0	GraphPad Software, LLC	RRID: SCR_002798https://www.graphpad.com
DESeq2	Bioconductor	RRID:SCR_015687
Bowtie2		RRID:SCR_016368
Gene set enrichment analysis		RRID:SCR_003199
ClusterProfiler		RRID:SCR_016884
Metascape	https://metascape.org/	RRID:SCR_016620
TrimGalore	https://github.com/FelixKrueger/TrimGalore	RRID:SCR_011847

**Other**		

DMEM/F-12	Gibco	Catalog number: 31330095
RPMI-1640	Gibco	Catalog number: 61870044
Fetal Bovine Serum (FBS)	Gibco	Catalog number: 11573397
Penicillin-Streptomycin	Gibco	Catalog number: 15140122
Trypsin EDTA (0.25%)	Gibco	Catalog number: 25200072
Opti-MEM Reduced Serum media	Thermofisher	Catalog number: 31985062
4-12% Bolt BisTris SDS-PAGE gel	ThermoFisher Scientific	Catalog number: NW04120BOX
LDS Sample Buffer	ThermoFisher Scientific	Catalog number: BP0007
MES SDS Running Buffer	ThermoFisher Scientific	Catalog number: NP0002
NuPAGE Reducing Agent	ThermoFisher Scientific	Catalog number: NP0009
iBright Prestained Protein Ladder	ThermoFisher Scientific	Catalog number: LC5615
iBlot™ 2 Transfer Stack, nitrocellulose	ThermoFisher Scientific	Catalog number: IB23001
Pierce Fast Blocking Buffer	ThermoFisher Scientific	Catalog number: 37575
PBS	Gibco	Catalog number: AM9625
TWEEN 20	Millipore-Aldrich	Catalog number: CAS-9005-64-5
SuperSignal™ West Pico PLUS Chemiluminescent Substrate	Thermo Fisher Scientific	Catalog number: 34580
Dynabeads Protein G	ThermoFisher	Catalog number: 10004D

### Resource availability

#### Lead contact

Further information and requests for resources and reagents should be directed to and will be fulfilled by the Lead Contact, Dr. Fahad Ali (fahad.ali@mbru.ac.ae).

#### Materials availability

This study generated new FOXC1 KO cell lines in four parental TNBC cell lines which can be requested by contacting Dr. Fahad Ali. Mohammed Bin Rashid University would require an MTA to be signed for sharing of these cell lines.

#### Data and code availability

• All raw and processed ChIP-seq and RNA-seq sequencing data generated in the current study have been submitted to the NCBI Gene Expression Omnibus (GEO; https://www.ncbi.nlm.nih.gov/geo/) and have been made publicly accessible under accession number GEO:GSE213841. The mass spectrometry proteomics data have been deposited to the ProteomeXchange Consortium via the PRIDE partner repository^47^ with the dataset identifier PRIDE:PXD053261. Raw data from Figure 1 was deposited on Mendeley at Mendeley Data: https://doi.org/10.17632/b4c28szwyn.1. All other data needed to evaluate the conclusions in the paper are present in the paper, in the above GEO project and/or the Supplementary Materials and tables. Publicly available data used in this study was downloaded from GEO. The accession numbers for the datasets are listed in the key resources table.
• This paper does not report original code.
• Any additional information required to reanalyze the data reported in this paper is available from the lead contact upon request.

### Experimental model and study participant details

#### Cell lines

Cell lines used in this study include four female TNBC cell lines BT549 (RRID:CVCL_1092), Hs578t (RRID:CVCL), MDA-MB-231 (RRID:CVCL_0062) and MDA-MB-468 (RRID:CVCL_0419). Cell lines generated in this study include four FOXC1_Knockout clones generated from the above parental TNBC cell lines (BT549_FOXC1_KO, Hs578t_FOXC1_KO, MDA-MB-231_FOXC1_KO, MDA-MB-468_FOXC1_KO) isolated from single colonies and verified to possess homozygous, frameshifting indels in the FOXC1 open reading frame. Refer to the key resources table and method details for information on the source and generation of each cell line and to the method details below for culture conditions.

### Method details

#### Cell lines and culture maintenance

TNBC cell lines, BT-549 (HTB-122), Hs578t (HTB-126), MDA-MB-231 (HTB-26), and MDA-MB-468 (HTB-132) were purchased from AddexBio (San Diego, CA). Hs578t and MDA-MB-231 were maintained in DMEM/F-12 (Gibco, ThermoFisher Scientific), whereas BT-549 and MDA-MB-468 were maintained in RPMI 1640 (Gibco) in a 5% CO_2_ incubator at 37°C. The medium was supplemented with 10% Fetal Bovine Serum (FBS) (South American origin, Gibco) and 1% Penicillin-streptomycin (Gibco).

#### CRISPR gene editing of cell lines to create FOXC1 knockout

Knockout of FOXC1 was performed using CRISPR/Cas9. Two predesigned TrueGuide synthetic sgRNA (CRISPR684819_SGM: GAACGCCCCGGACAAGAAGA or CRISPR684834_SGM: GACAAGAAGATCACCCTGAA) (ThermoFisher Scientific, Waltham, USA) targeting the single exon in FOXC1 gene were complexed with Truecut Cas9 protein v2 (ThermoFisher Scientific) and used to transfect cells. The efficiency of genomic cleavage at the FOXC1 gene loci was determined using the GeneArt Genomic cleavage detection kit (ThermoFisher Scientific), and wells with the highest cleavage efficiency were selected. Single cells were plated into 6 x 96 wells and incubated for 4–6 weeks in an Incucyte Zoom system (Essen Biosciences, Ann Arbor, USA) with images taken every 24 h. Clonal populations derived from single cells were selected following limited dilution of CRISPR-edited libraries. Potential clones were verified to have originated from a single cell and passaged. An aliquot of cell suspension was used to verify homozygous editing at the FOXC1 locus using the Takara Guide-It Genotype Confirmation kit (Takara, Kusatsu, Japan). FOXC1 genomic region was amplified using primers (RR042: 5′ CCATGAGCGTGTACTCGCA and RR043: 5′ GCGCATCCAGGACATCAAGA) and sent for sanger sequencing (Macrogen, Seoul, South Korea). Sanger sequencing data were analyzed using ICE Analysis (2019. V3.0. Synthego, Redwood City, USA) to identify clones that were successfully edited for homozygous deletion in both FOXC1 alleles. Confirmation of FOXC1 knockout was performed using western blot analysis.

#### Western Blot analysis of FOXC1 protein expression

Western blotting was performed to confirm FOXC1 knockout in CRISPR-edited clones.^48^ A total of 75 μg of cell lysate (quantified using Pierce BCA Protein Assay Kit, ThermoFisher Scientific) was used to ensure the absence of FOXC1 protein expression in the KO clones. Protein lysates were mixed with LDS sample buffer and Reducing Agent (ThermoFisher Scientific) and heated at 85°C for 10 min, before being centrifuged for 2 min at 13,000 rpm. Soluble lysate fractions were loaded onto precast 4–12% Bolt BisTris SDS-PAGE gel (ThermoFisher) and electrophoresed using Bolt MES SDS Running Buffer (ThermoFisher) and transferred onto a nitrocellulose membrane using the iBlot dry transfer system (ThermoFisher). The membrane was blocked using Pierce Fast Blocking buffer (ThermoFisher) supplemented with 5% non–fat milk for 1 h at room temperature and incubated overnight at 4°C with anti-FOXC1 (1:1000; ab227977 Abcam, Cambridge, UK) or anti-β-actin (1:10000; ab6276, Abcam) as an internal control, diluted in blocking buffer with 2% non-fat milk. After four washes in 1x PBST, blots were incubated for 1 h with HRP-conjugated secondary anti-rabbit or anti-mouse (1:10000; Abcam), respectively. Blots were exposed to Pierce SuperSignal West Pico PLUS Chemiluminescent Substrate and visualized using the Azure 600 Imager (Azure Biosystems, Dublin, CA, USA).

#### Growth and proliferation assay

An equal number of cells (5000-15,000 depending on the cell line) were seeded onto 24-well tissue culture-treated plates, and three wells were trypsinized and counted every 24 h. Cell viability was analyzed by mixing equal volumes of resuspended cells with 0.4% trypan blue (Biorad, Hercules, CA, USA). After the addition of trypan blue, the cells were immediately analyzed, and live cells were quantified using the CellDrop automated cell counter (Denovix, Wilmington, USA).

#### Colony forming assay

Cells were seeded into 6-well tissue culture plates (150–300 cells/well depending on cell line) and grown for 14 days. The cells were fixed with acidified methanol and stained with 0.05% crystal violet solution. Colonies were imaged in white light using the A600 Imager (Azure Biosystem). Colonies were manually counted in three biological replicates, each with three technical replicates.

#### Invasion and migration assays

Migration and invasion assays were performed in Transwell chambers containing 8-μm pore membranes (6.5 mm Transwell with 8.0 μm Pore Polyester Membrane Insert, Costar, Corning Incorporated, Corning, USA). In brief, approximately 100,000–200,000 cells resuspended in 200 μL serum-free media were seeded into the upper chamber, uncoated or coated with a matrix, Geltrex LDEV-Free, hESC-Qualified, Reduced Growth Factor Basement Membrane Matrix (Gibco) using 100 μL of 1:5 diluted matrix per insert and allowed to set for 1 h at 37°C prior to adding the cell suspension. Growth medium containing 10% FBS was added to the lower chamber, and the plate was incubated for 20 h at 37°C with 5% CO_2_. The inner chamber of the Transwell was gently wiped using a moist Q-tip, and cells on the bottom of the Transwell insert were fixed with formaldehyde, stained with 0.05% crystal violet (dissolved in a 7:1Methanol: Acetic acid), and imaged using an inverted microscope (Olympus) at 10X magnification. Four representative images were taken for each well, and the percentage of stained cells present was calculated using ImageJ. Two biological replicates were performed in four technical replicates for each sample along with a no FBS control.

#### Propidium iodide staining and cell cycle analysis using flow cytometry

Cells were harvested from 10 cm tissue culture treated plates and fixed in 70% ethanol, washed twice with cold 1 × PBS, and then incubated at 37°C in a staining solution containing 100 mM Tris-HCl, 150 mM NaCl, 0.1% Igepal, 1 mM CaCl_2_, 0.5 mM MgCl_2_ and 20 μg/mL RNase A (ThermoFisher). Propidium Iodide (ThermoFisher) was added to a final concentration of 1.5 μM to an equal number of RNAse-treated cells and incubated overnight at 4°C. Samples were strained using a 40 μM cell strainer prior to being detected on a FACSaria III flow cytometer (Becton-Dickinson, Franklin Lakes, USA), and the percentages of cells within each phase of the cell cycle were analyzed using FlowJo cell cycle module.

#### RNA-seq

RNA was isolated from the following eight samples in three biological replicates: BT-549, BT-549_FOXC1_KO, Hs578t, Hs578t_FOXC1_KO, MDA-MB-231, MDA-MB-231_FOXC1_KO, MDA-MB-468 and MDA-MB-468_FOXC1_KO using RNeasy mini kit (Qiagen, Hilden, Germany), with on-column DNase treatment to remove genomic DNA.

Briefly, total RNA was isolated from the following eight samples in three biological replicates: BT-549, BT-549_FOXC1_KO, Hs578t, Hs578t_FOXC1_KO, MDA-MB-231, MDA-MB-231_FOXC1_KO, MDA-MB-468 and MDA-MB-468_FOXC1_KO using RNeasy mini kit (Qiagen, Hilden, Germany), with on-column DNase treatment to remove genomic DNA.

In detail, RNA (1 μg) from BT-549, BT-549_FOXC1_KO, MDA-MB-468, and MDA-MB-468_FOXC1_KO was used to prepare libraries using the Illumina stranded mRNA library prep kit. RNA was quantified using Qubit BR RNA kit (Invitrogen), and the integrity of RNA was determined using Tape station (Agilent). Strand-specific mRNA sequencing libraries were prepared using Illumina Stranded mRNA Library Prep Kit (Illumina), following the manufacturer’s procedure. The fragments were amplified and quantified with KAPA quantification kit (Roche), and the library size was determined using Tape station (Agilent). Libraries were sequenced on the NovaSeq 6000 in a 150 bp paired-end run to a depth of at least 20 million reads per library. RNA from Hs578t, Hs578t_FOXC1_KO, MDA-MB-231, and MDA-MB-231_FOXC1_KO were similarly sequenced at Macrogen (Seoul, Korea) with libraries generated using the TruSeq Stranded mRNA Library Prep Kit and sequenced on an Illumina platform using 101 bp paired-end reads on the Illumina platform. Approximately 40–60 million reads were achieved for each sample. Reads were trimmed using TrimGalore 0.6.4 (https://github.com/FelixKrueger/TrimGalore) to remove sequencing adaptors and poor-quality base calls, using a minimum Phred score cut-off of 20. Trimmed reads were aligned to the hg38 genome with STAR_2.6.1day (Anders and Huber, 2010) and quantified using the quantMode option. Genes with read counts of at least 10 in all biological replicates per condition were retained for downstream processing. Read counts were normalized with DESeq2 rlog transformation for PCA plots, selecting the 500 most variable genes. DEGs were identified using DEseq2 1.30.0 (Love et al., 2014) padj <0.05 and no logfold change cut-off, with the contrasts FOXC1 KO vs. FOXC1 WT for each cell line. Pearson’s correlation coefficients were calculated based on gene counts for each replicate set, and biological replicates with a correlation <0.95 were excluded from further analysis, resulting in 3 biological replicates per group, except for MDA-MB-231_KO, which was left with 2 replicates after quality control.

#### Chromatin immunoprecipitation sequencing (ChIP-seq)

ChIP-seq was performed as previously described^48^ using FOXC1 antibody generated against a recombinant FOXC1 protein deleted for the forkhead domain (Δ69-178),^19^ in order to reduce non-specific binding by other forkhead-domain containing proteins. Briefly, 20 x 10^6^ cells (3 × 15 cm confluent dishes) were fixed with 1% formaldehyde for 10 min and quenched with 100 mM Glycine. Cells were washed twice with PBS with 100 mM PMSF (Roche), collected by scraping and pelleted by centrifugation at 2000 x g for 5 min at 4°C. Pellets were stored at −80°C. For ChIP, nuclei isolation was performed by resuspending cell pellet in 3 mL (1 mL per 15 cm dish) of cold lysis buffer 1 (LB1: 50 mM Hepes-KOH pH 7.5, 140 mM NaCl, 1 mM EDTA, 10% (v/v) glycerol, 0.5% NP-40/Igepal CA-630, 0.25% Triton X-100) and rotated at 4°C for 10 min. Lysates were cleared by centrifugation at 2,000 x g for 5 min at 4°C and pelleted nuclei were resuspended 3 mL of cold LB2 (10 mM Tris-HCl pH 8.0, 200 mM NaCl, 1 mM EDTA, 0.5 mM EGTA) and the samples were rotated at 4°C for 10 min. Samples were cleared by centrifugation at 2000 x g for 5 min at 4°C. Purified nuclear pellets were resuspended in 300 μL of LB3 (10 mM Tris-HCl pH 8.0, 100 mM NaCl, 1 mM EDTA, 0.5 mM EGTA, 0.1% (w/v) sodium deoxycholate, 0.5% (v/v) N-lauroylsarcosine) for sonication using a Bioruptor pico sonicator (Diagenode, Denville, NJ, USA). Sonication efficiency was measured by agarose gel electrophoresis to ensure that de-crosslinked DNA was fragmented to an average size 100–300 bp. 10 μg of FOXC1 antibody bound to Protein-G coupled Dynabeads (Invitrogen) was used to precipitate bound DNA from 300 to 500 μg of sheared chromatin by overnight incubation at 4°C with rotation. Beads were washed 6 times in RIPA buffer (50 mM HEPES pH 7.6, 0.5 M LiCl, 1 mM EDTA pH 8.0, 0.7% sodium deoxycholate, 1% NP-40) followed by 1 wash using TE buffer (10 mM Tris-HCl, 1 mM EDTA, pH 8). Cross-linking was reversed by adding 200 μL ChIP elution buffer (50 mM TrisHCl, pH 8.0, 10 mM EDTA and 1% SDS) and incubating overnight at 65°C. Samples were treated with 100 μg/mL RNAse A (Qiagen) for 1 h at 37°C followed by Proteinase K digestion for 1 h at 37°C. ChIP DNA was eluted in 25 μL of nuclease-free TE buffer, pH8, purified using the Qiagen PCR purification kit (Qiagen, Hilden, Germany) and quantified with the Qubit dsDNA BR assay kit (Invitrogen). ChIP sample libraries were generated from three biological replicates, and the respective input samples were pooled into one sequencing library, generated using the NEBNext Ultra II DNA Library Prep Kit (Illumina, San Diego, CA, USA). The amplified libraries were quantified using the KAPA quantification kit (Roche, Basel, Switzerland), and the library size was determined using Tape station (Agilent, Santa Clara, CA, USA). Libraries were sequenced on the NovaSeq 6000 in a 150 bp paired-end run to a depth of at least 25 million mapped reads per sample. Reads were trimmed using TrimGalore 0.6.4 (https://github.com/FelixKrueger/TrimGalore) to remove sequencing adaptors and poor-quality base calls, using a minimum Phred score cut-off of 20. Trimmed reads were aligned to the hg38 genome with Bowtie2 2.4.1 (Langmead and Salzberg, 2012). Unmapped reads, improperly paired reads, reads with> 4 mismatches, or insert size >2000 bp were removed. BigWig files were generated by combining bam files from individual biological replicates and subsampling to 160 million reads before converting to BigWig format. FOXC1 peaks were called for pooled replicates-input pairing, using Macs2 2.2.7.1 (Zhang et al., 2008) in narrowpeak mode, using the following options: -f BAMPE -g hs --SPMR --qvalue 0.05 --keep-dup all. Peaks with a less than 5-fold enrichment over background, peaks in centromere regions, and peaks in hg38 blacklisted regions were removed, to generate a list of high-confidence FOXC1 peaks for each cell line. FOXC1 peaks were linked to predicted target genes within 50 kb, based on significant differential expression (FOXC1 KO vs. FOXC1 WT) of the potential target gene from the RNA-seq data. If no genes within 50 kb were significantly differentially expressed, the closest gene was assigned as the regulatory target. Significantly enriched motifs were identified using Homer findMotifsGenome.pl with high-confidence peaks for each cell line as the input and the options -size 100 -mask. Consensus motifs were plotted with motifStack in R.

#### Bioinformatic analysis

PCA and sample-to-sample distance heatmaps were plotted using DiffBind normalized count data using default DiffBind settings for ChIP-seq and VST normalized data in DESeq2 RNA-seq data. Gene set enrichment analysis was performed with fgsea 1.160 in R using Reactome Pathway Database gene sets, or GO BP gene sets. Pathways with an adjusted *p*-value <0.05 were considered significantly enriched. Gene set overrepresentation analysis was performed using ClusterProfiler 3.18.1 in R. GO BP or Reactome gene sets reaching adjusted *p*-value (BenjaminiHochberg adjusted) < 0.05 were considered significant. Pathway enrichment from various gene lists was performed using Metascape^49^ or ShinyGO.^22^ Kaplan–Meier survival curves were generated using gene expression data from public microarray datasets using kmplottter.^18^ All tumors (Figure 5H), or tumors classified as ER- (IHC), PR-(IHC) and HER2 negative (array) (Figures 2D and S5) were evaluated for relapse free survival benefits, split using auto-selected cut-off values for gene expression and Jetset best probe set options.

#### Rapid immunoprecipitation of endogenous proteins (RIME)

RIME was performed as from two biological replicates using FOXC1 antibody^19^ and matched IgG negative controls (ab6706, Abcam). Briefly, 2 × 15 cm confluent plates (approximately 10 x 10^6^) cells were washed with PBS, and incubated with freshly prepared 2 mM DSG (disuccinimidyl glutarate, Thermoscientific) dissolved in PBS for 20 min, followed by crosslinking with 1%, formaldehyde for 10 min at room temperature. Crosslinking was quenched by the addition of 100 mM Glycine, following which the cells were rinsed with ice-cold PBS +100 mM PMSF (Roche), scraped using a silicone scraper and collected by centrifugation at 2,000 x g for 5 min at 4°C. Pellets were stored at −80°C. The cell pellets were lysed as described for chromatin immunoprecipitation and incubated with 5 μg of FOXC1 or IgG (Abcam, ab6706) antibody and incubated overnight at 4°C with rotation. Antibody-bound Dynabeads (Invitrogen) were washed 10 times in RIPA buffer (50 mM HEPES pH 7.6, 0.5 M LiCl, 1 mM EDTA pH 8.0, 0.7% (w/v) sodium deoxycholate, 1% (v/v) NP-40) followed by two washes in 100 mM Ammonium bicarbonate solution to remove detergents and salts. The washed beads were flash frozen in liquid nitrogen. On-bead tryptic digest for LC/MS-MS and mass spec identification of peptides was performed by the proteomics core facility at Cancer Research UK Cambridge Institute. Proteins were considered as interactors when high-confident peptides were identified in both replicates and when none of these peptides were identified in matched IgG-negative controls.

#### Cloning and over-expression of FOXC1 and NR2F2 in Bt-549

The coding sequence of FOXC1 was amplified from FLAG-FOXC1 (a gift from Stefan Koch, Addgene plasmid # 153114) using primers RR022 (5′ CCCTCGTAAAGAATTCGCCACCATGGATTACAAGGACGACG 3′) and RR023 (5′ GAGGTGGTCTGGATCCTCAAAACTTGCTACAGTCGTAGA3′) and ligated into pLVX-TetOne-Puro (Takara) using the In-Fusion HD Cloning Kit (Takara). Plasmid was verified by sequencing. Plasmid pLenti-CMVSP6-NR2F2-SV40-PURO was a gift from Nathan Lawson (Addgene plasmid # 138361). Plasmids were purified using EndoFree Plasmid Maxi Kit (Qiagen, Germany). Transfection was performed using 14 μg of each plasmid and Lipofectamine 3000 (Invitrogen), as per manufacturers protocol, in 10 cm Petri dish with 70% confluent Bt549. FOXC1 expression was induced using 1 μg/mL of Doxycyclin for 48 h prior to harvesting the cell pellet.

#### Co-immunoprecipitation of FOXC1 and NR2F2

Bt-549 cells transfected with plasmids overexpressing FOXC1 and NR2F2 in 10 cm Petri dish was harvested and the cell pellet was resuspended in 100 μL of RIPA lysis buffer (ThermoFisher Scientific) containing 100 mM PMSF and 1X Protease Inhibitor cocktail (ThermoFisher Scientific). Cell lysate was sonicated for 10 cycles (Bioruptor Pico, Diagenode), centrifuged and the supernatant was incubated overnight with Protein-G beads coupled with 5 μg of anti-NR2F2 (sc271265x, Santacruz Biotech, Dallas, Texas) or anti-IgG (Ab6706, Abcam). Unbound protein was washed using RIPA Buffer and protein complexes eluted using 4X Bolt LDS Sample buffer supplemented with 1X Reducing agent (ThermoFisher Scientific). Western blotting was performed as described above.

### Quantification and statistical analysis

Barplots in this study represent mean ± standard deviation from three independent biological replicates, unless otherwise stated. *p*-value was computed using multiple unpaired t tests in Prism GraphPad software and adjusted *p*-values representing differential regulation in RNA-seq data were calculated using DESeq2. *p*-value <0.05 was considered significant. Details of statistical analysis can be found in the figure legends.

## Supplemental information

Document S1. Figures S1–S10, Data/Methods S1 and S2, and Table S1

Table S2. FOXC1 peaks in five TNBC clusters, related to Figure 3

Table S3. Summary of FOXC1 cofactors identified in RIME experiments, related to Figure 4

Table S4. 164 core gene targets of FOXC1 in breast cancer, related to Figure 5
