# Supplementary material for: Conserved role of FOXC1 in TNBC is parallel to FOXA1 in ER+ breast cancer
Source: iScience. 2024 Jul 14;27(8):110500. doi: 10.1016/j.isci.2024.110500 (PMC11338131; doi:10.1016/j.isci.2024.110500)
Supplement: Document S1. Figures S1–S10, Data/Methods S1 and S2, and Table S1 [file mmc1.pdf]

iScience, Volume *27*

Supplemental information

Conserved role of FOXC1 in TNBC is parallel to FOXA1 in ER+ breast cancer

Revathy Ramachandran, Shakhzada Ibragimova, Laura M. Woods, Tamader AlHouqani, Roshna Lawrence Gomez, Fabrizio Simeoni, Mahmood Y. Hachim, Tim C.P. Somervaille, Anna Philpott, Jason S. Carroll, and Fahad R. Ali

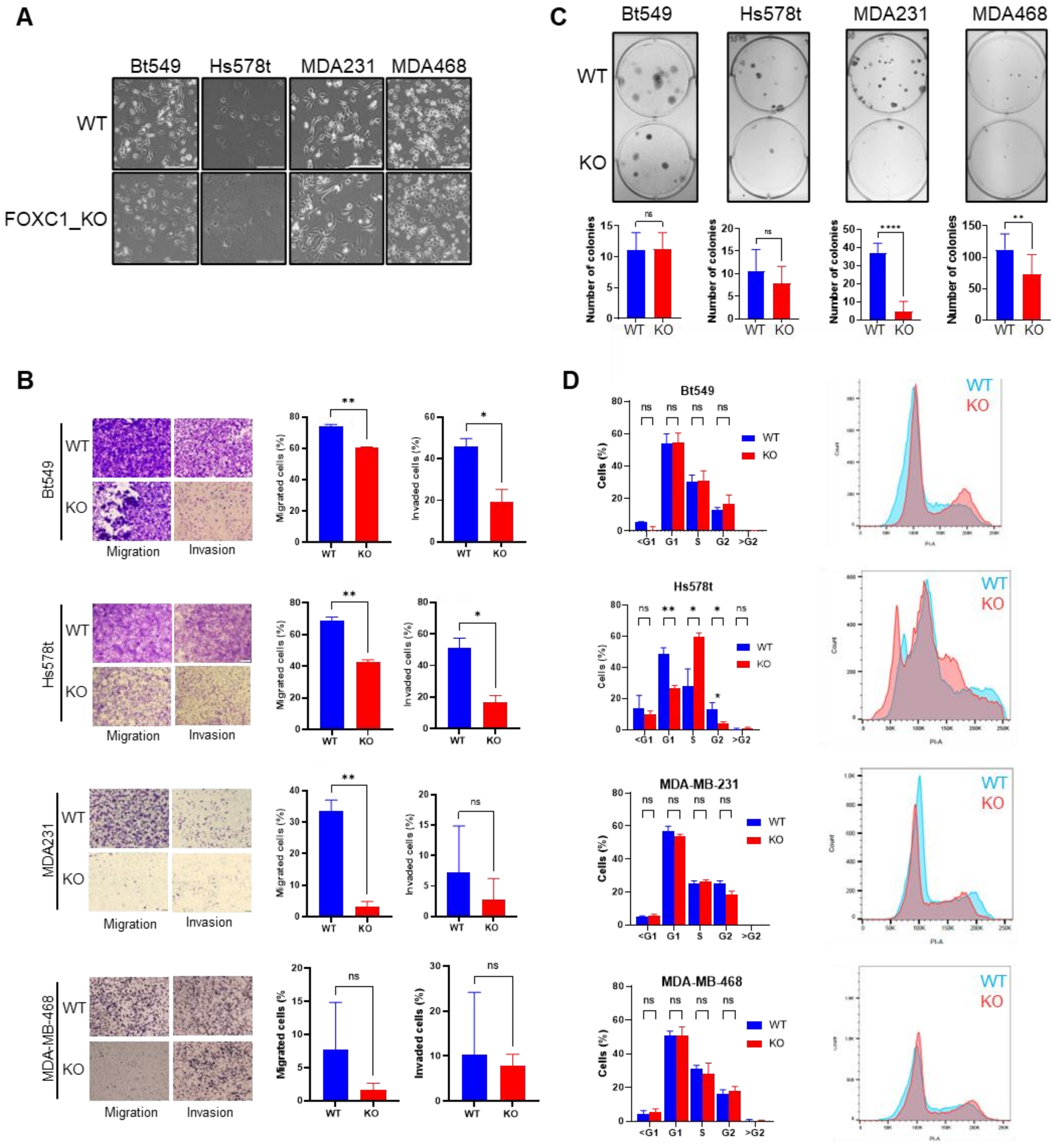

Fig. S1. Effect of FOXC1 knockout on tumor-associated phenotypes (Related to Figure. 1).

(A) Phenotypic changes in cell morphology upon FOXC1 KO, at 10X magnification, phase contrast microscopy. Scale bar at bottom right represents 200 µm. **(B)** Migration (left) and Invasion (right) assays using a transwell chamber alone (left) or coated with matrigel (right) showing number of migrated/invaded cells upon loss of FOXC1. Data represent mean and standard deviation (SD) from two biological replicates. **(C)** Colony-forming assays showing the number of colonies in FOXC1 KO versus parental lines. Cells were grown in 6-well plates for 14 days prior to crystal violet staining. Bar graphs

(bottom panel) display the mean and standard deviation (SD) from three biological replicates, each with three technical replicates. **(D)** Bar charts (left panel) showing quantification of percentage of cells in <G1, G1, S or G2 (mean±SD) from three biological replicates in parental (WT, blue) or FOXC1_KO (KO, red) cell lines. Asterisk denotes significant digits in p-value derived from unpaired t-tests. * < 0.05, *** < 0.005. Representative cell cycle profiles between WT (blue) or FOXC1_KO (red) are shown on the right.

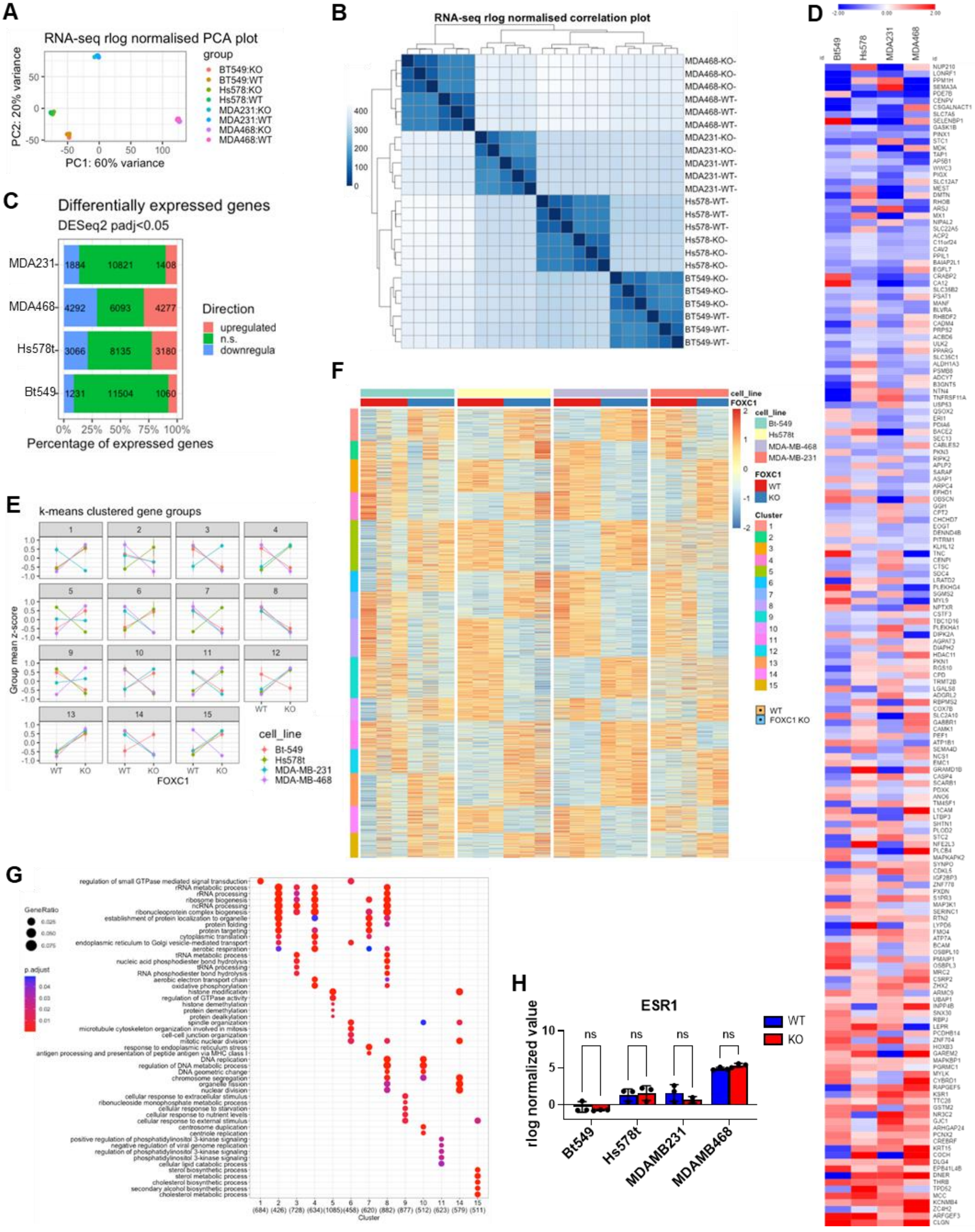
A
RNA-seq rlog normalised PCA plot
PC1: 60% variance
PC2: 20% variance
group
BT549:KO
BT549:WT
Hs578:KO
Hs578:WT
MDA231:KO
MDA231:WT
MDA468:KO
MDA468:WT
B
RNA-seq rlog normalised correlation plot
C
Differentially expressed genes
DESeq2 padj<0.05
MDA231 1884 10821 1408
MDA468 4292 6093 4277
Hs578t 3066 8135 3180
Bt549 1231 11504 1060
Percentage of expressed genes
Direction
upregulated
n.s.
downregula
D
E
k-means clustered gene groups
Group mean z-score
FOXC1
cell_line
Bt-549
Hs578t
MDA-MB-231
MDA-MB-468
F
cell_line
FOXC1
Cluster
WT
KO
FOXC1 KO
G
GeneRatio
p.adjust
Cluster
H
ESR1
rlog normalized value
WT
KO
ns
Bt549
Hs578t
MDAMB231
MDAMB468

Fig. S2. RNA-seq analysis of TNBC cell lines and FOXC1_KO clones (Related to Figure 1).

(A)Principal component analysis of TNBC cell lines "WT" and the FOXC1_KO "KO" using rlog transformed data for the top 500 variable genes reveals greater similarity between gene expression signatures of Hs578t and BT-549 cell lines, as compared to MDA-MB-231. **(B)** Correlation plot of biological replicates in each sample shows clustering of KO with respective parental cell lines. **(C)** Number of significantly (adjusted p-value < 0.05) differentially expressed genes (DEGs) identified in each WT vs FOXC1_KO pair. **(D)** Heatmap of log fold change (Log2FC) values from normalized mRNA expression of the 172 genes that were significantly (padj value <0.05) differentially expressed between WT and FOXC1_KO of the four TNBC cell lines in RNA-seq analysis. **(E)** Representation of the eight states for identifying patterns gene expression changes across the WT and FOXC1_KO in the four cell lines revealing 15 patterns of expression. **(F)** Heatmap depicting change in gene expression within 15 unsupervised k-means clusters in the previous panel. **(G)** Enrichment of GO BP terms within each identified K-means cluster from panel D. Note that clusters 12 and 13 did not have any significantly enriched GO terms. **(H)** Bar plots showing the normalised expression of ESR1 mRNA in parental (Blue, WT) and FOXC1_KO (Red, KO) in TNBC cell lines. Statistical significance determined using DESeq2 (ns = not significant).

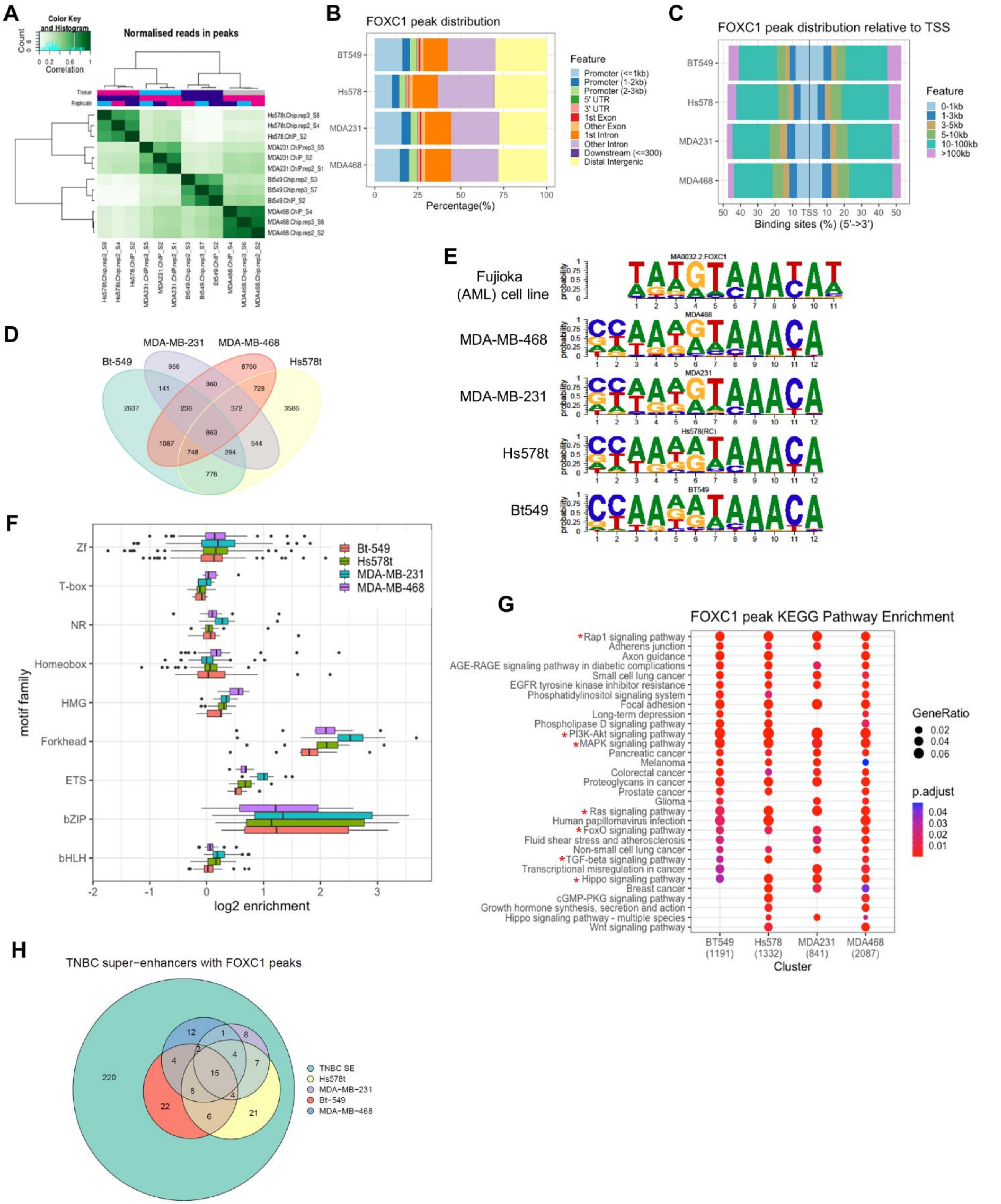

Fig. S3. Analysis of FOXC1 binding sites (Related to Figure 2).

(A) Pearson's correlation of normalized reads in peaks in individual biological replicates of ChIP-seq from four different TNBC cell lines. **(B)** Distribution of FOXC1 peaks in features in the respective cell

lines and **(C)** Distribution of FOXC1 peaks relative to the TSS. **(D)** Venn diagram depicting overlap of FOXC1 peaks, with 863 peaks conserved between all four cell lines. **(E)** Top de novo motifs in high confidence FOXC1 peaks for each cell line, aligned to FOXC1 consensus motif identified previously [1]. **(F)** TF motifs enriched within 200 bp of a FOXC1 peak showing enrichment of main families of motif. **(G)** KEGG terms significantly enriched among list of genes associated with peaks in each cell line. Red asterisks highlight important signaling pathways. **(H)** Euler plot showing number of FOXC1 peaks in TNBC-specific super-enhancers identified in [2].

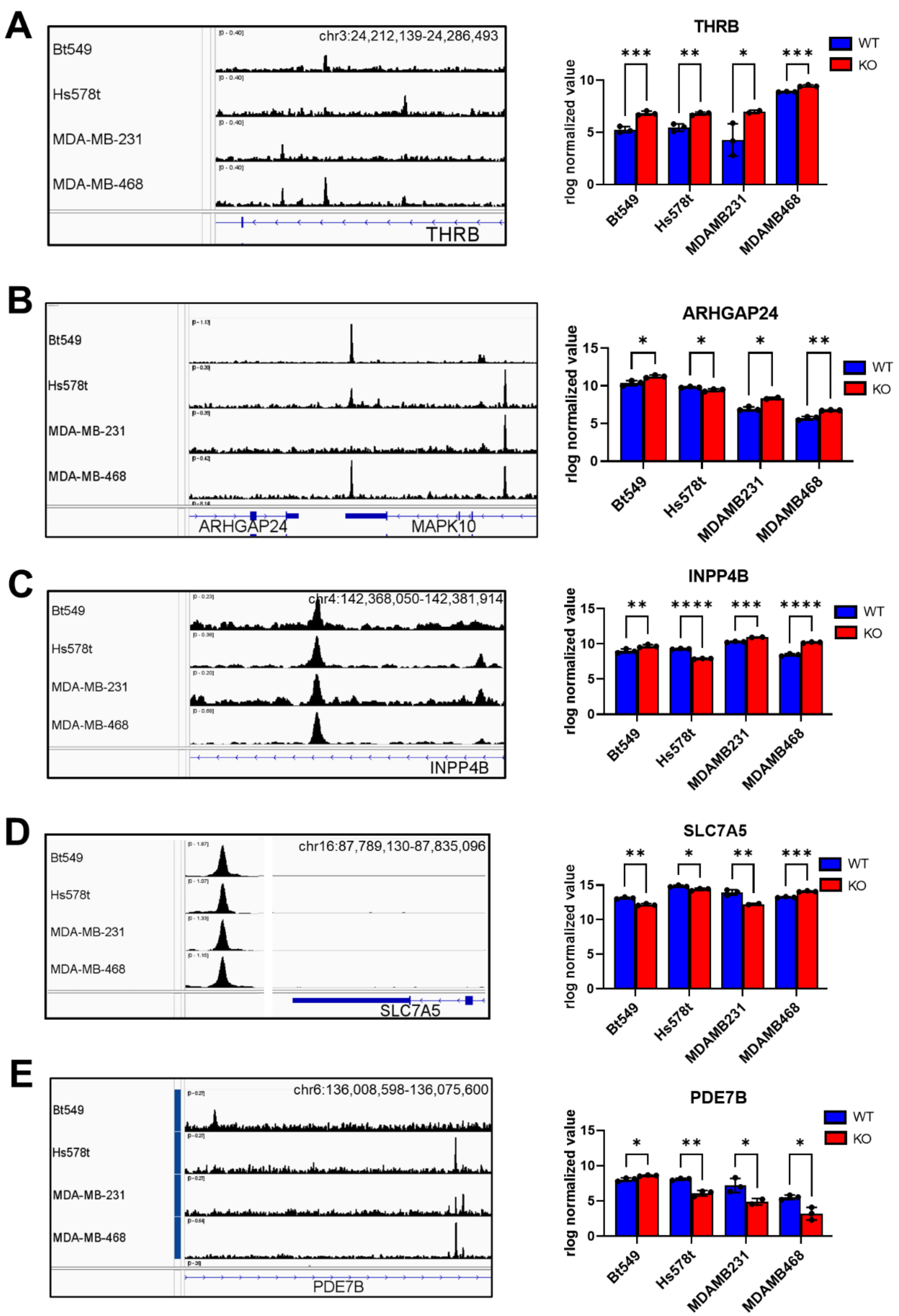
A
Bt549
Hs578t
MDA-MB-231
MDA-MB-468
chr3:24,212,139-24,286,493
THRB
THRB
rlog normalized value
WT
KO
***
**
*
***
Bt549
Hs578t
MDAMB231
MDAMB468
B
Bt549
Hs578t
MDA-MB-231
MDA-MB-468
ARHGAP24
MAPK10
ARHGAP24
rlog normalized value
WT
KO
*
*
*
**
Bt549
Hs578t
MDAMB231
MDAMB468
C
Bt549
Hs578t
MDA-MB-231
MDA-MB-468
chr4:142,368,050-142,381,914
INPP4B
INPP4B
rlog normalized value
WT
KO
**
****
***
****
Bt549
Hs578t
MDAMB231
MDAMB468
D
Bt549
Hs578t
MDA-MB-231
MDA-MB-468
chr16:87,789,130-87,835,096
SLC7A5
SLC7A5
rlog normalized value
WT
KO
**
*
**
***
Bt549
Hs578t
MDAMB231
MDAMB468
E
Bt549
Hs578t
MDA-MB-231
MDA-MB-468
chr6:136,008,598-136,075,600
PDE7B
PDE7B
rlog normalized value
WT
KO
*
**
*
*
Bt549
Hs578t
MDAMB231
MDAMB468

Fig. S4. Tumor suppressors and Oncogenes among the conserved targets of FOXC1 (Related to Figure 2).

ChIP-seq traces and rlog normalized mRNA values from RNA-seq of selected FOXC1 conserved targets **(A)** THRB, Thyroid hormone receptor TRβ, a known tumor suppressor in TNBC, whose high expression correlates with better survival [3], forms heterodimers with RXR upon binding the agonist T3, and promotes tumor suppressive pathways [4]. TRβ also inhibits the expression of RUNX2, an oncogene in TNBC [5]. In our data, FOXC1 directly regulates the expression of THRB, RXRA, and RUNX2 in most cell lines **(B)** ARHGAP24, a tumor suppressor in many cancers suppresses cell invasion via regulating small GTPase RAC1 signaling in TNBC [6]. **(C)** INPP4B, is commonly inactivated in TNBC and promotes tumorigenesis by modulating degradation of EGFR and MET receptors [7]. Inactivation of INPP4B leads to higher tumor volume, sensitizing tumors to PI3K and MEK inhibitors, which is a potential targeted therapy approach for TNBC **(D)** SLC7A5, FOXC1 upregulates oncogeneSLC7A5 (LAT1), which is typically over-expressed in TNBC, and mediates uptake of Leucine in exchange for glutamine [8]. As Leucine is a regulator of mTORC1, SLC7A5 is speculated to promote proliferation via the Akt/mTORC1 pathway and is a target in preclinical trials. FOXC1 activates expression of SLC7A5 nearly 2-fold in all four cell lines via binding site within the first intron. **(E)** FOXC1 was seen to activate expression of PDE7B, which inhibits cell growth and tumor development by regulating cAMP concentration in TNBC [9]. Bar plots showing the normalised expression of mRNA in parental (Blue, WT) and FOXC1_KO (Red, KO) in TNBC cell lines. Statistical significance and p-value determined using multiple unpaired t tests using Graphpad Prism (Asterisk represents * = $p < 0.05$, ** = $p < 0.005$, ***= $p < 0.0005$, **** = $p < 0.00005$).

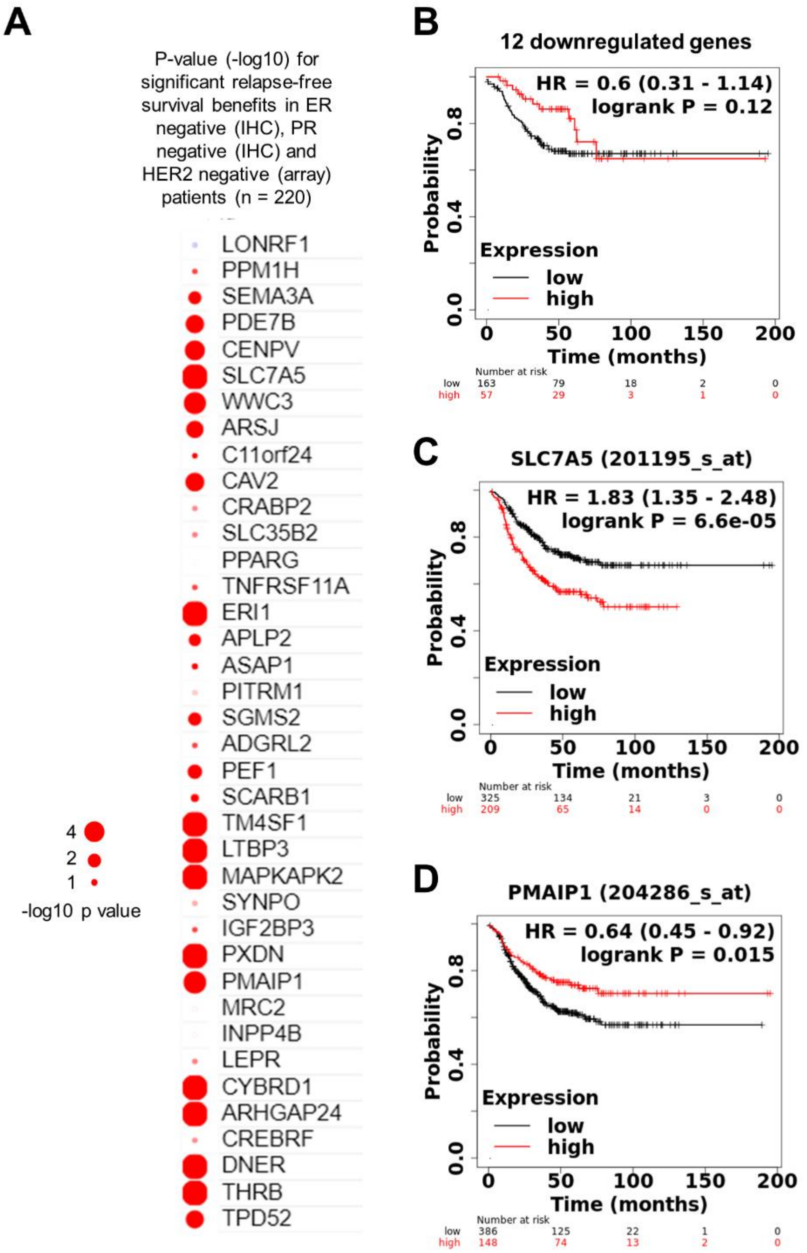

Fig. S5. Prognostic value of FOXC1 conserved targets (Related to Figure 2).

(A) Summary of (-log10) p-values of relapse-free survival (RFS) benefits in ER negative (IHC), PR negative (IHC) and HER2 negative (array) patients (n = 220) from kmplot.com (REF). **(B)** Kaplan-meier plot results of RFS in ER negative, PR negative and HER2 negative patients (n = 220) for a panel of 12 downregulated core gene targets of FOXC1. **(C & D)** Representative Kmplot analysis for oncogene SLC7A5 and tumor suppressor PMAIP1, indicating expression values correlate with RFS in ER negative, PR negative and HER2 negative patients (n = 220).

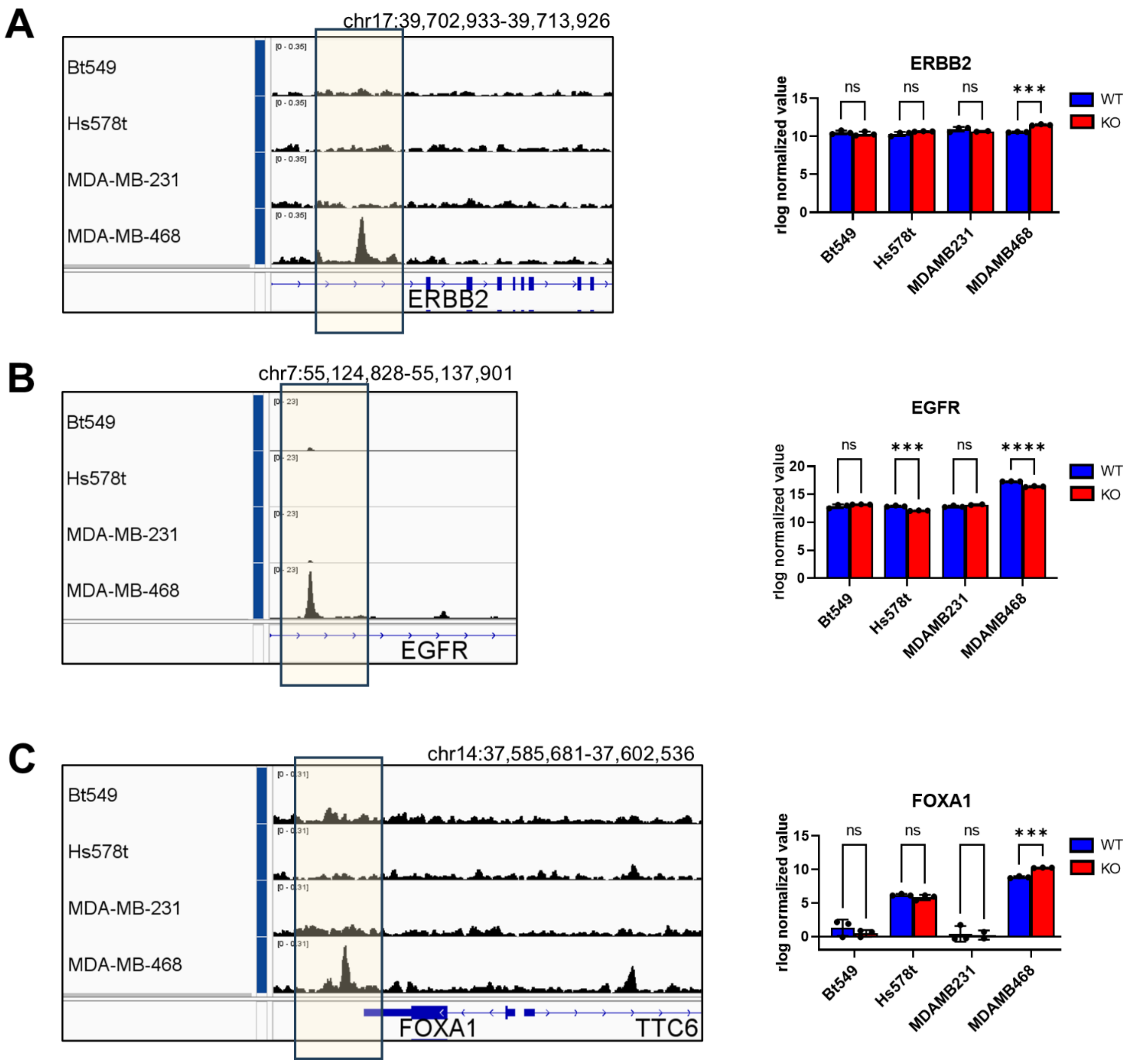

Fig. S6. Important genes in hormone/growth factor signaling in MDA-MB-468 regulated by FOXC1 (Related to Figure 2).

ChIP-seq traces and rlog normalized mRNA values from RNA-seq of **(A)** ERBB2 (HER2), **(B)** EGFR and **(C)** FOXA1 in all four cell lines, showing significant regulation only in MDA-MB-468. Bar plots show the normalised expression of mRNA in parental (Blue, WT) and FOXC1_KO (Red, KO) in TNBC cell lines. Statistical significance and p-value determined using multiple unpaired t tests using GraphPad Prism (ns = not significant, * = $p < 0.05$, *** = $p < 0.0005$, **** = $p < 0.00005$).

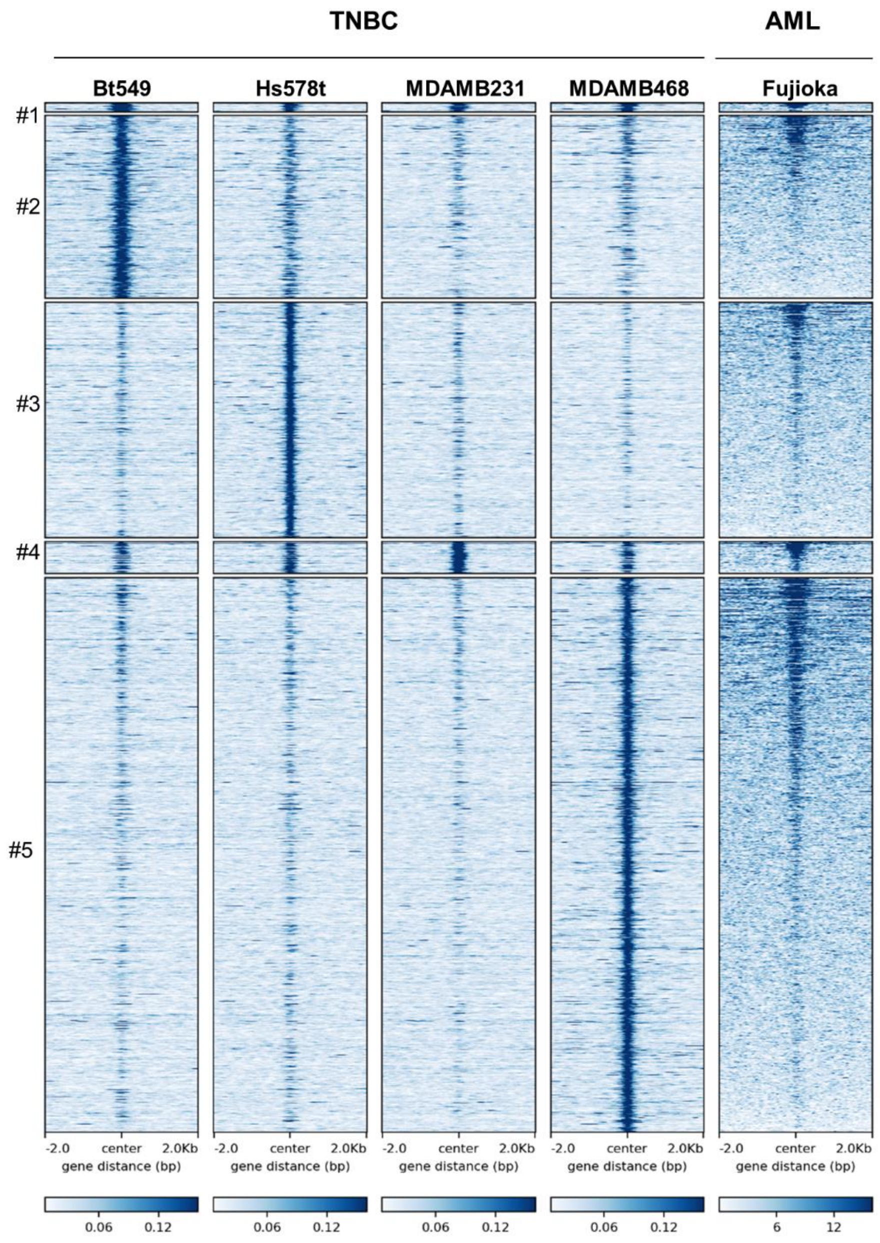

Fig. S7. Conservation of FOXC1 peaks in AML and TNBC (Related to Figure 3).

Overlap of FOXC1 ChIP-seq signal in Fujioka (AML) cell lines with the five FOXC1 peak clusters in TNBC from Fig. 3a.

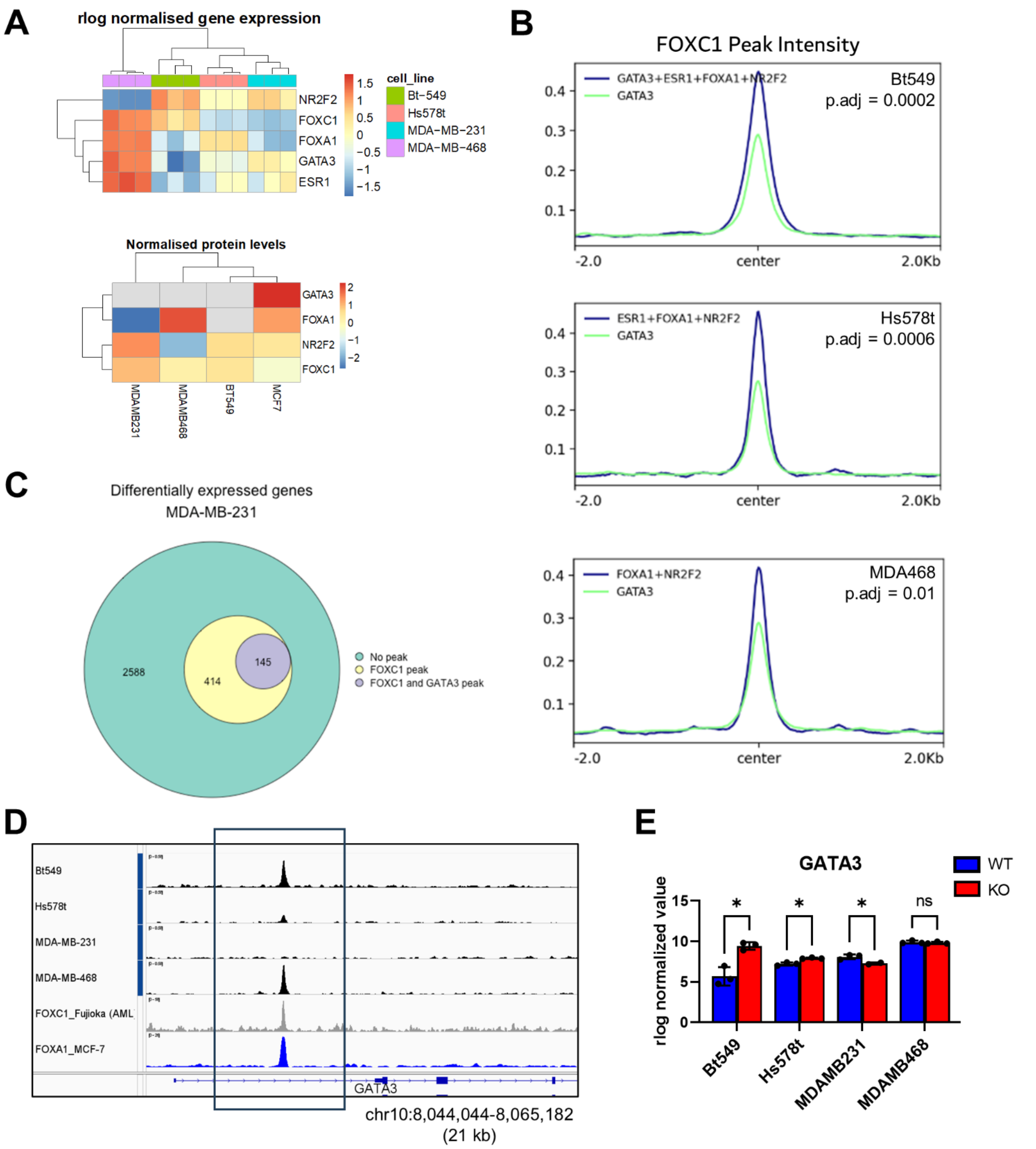

Fig. S8. FOXC1 and crucial Erα-associated transcription factors in ER+ breast cancer bind to similar sites (Related to Figure 5).

(A) Normalized gene expression from RNA-seq data (top panel) or protein expression from [10] of FOXC1 and crucial Erα-associated transcription factors (NR2F2, FOXA1, GATA3 and ESR1) in the four TNBC cell lines used in this study. **(B)** Average intensity of FOXC1 peaks in in Bt-549, Hs578t and

MDA-MB-468 (top to down) at sites that are erstwhile bound by a combination of NR2F2, FOXA1, ESR1 or GATA3 versus GATA3 alone. Adjusted p-value calculated using a Tukey post-hoc test. **(C)** Comparison of differentially expressed genes in MDA-MB-231 from this study, that have FOXC1 peak or both FOXC1 and GATA3 peak. **(D)** ChIP-seq traces near the GATA3 gene showing FOXC1 peaks in TNBC and AML cell lines and FOXA1 peaks in MCF-7. **(E)** Bar plots show the normalized expression of mRNA in parental (Blue, WT) and FOXC1_KO (Red, KO) in TNBC cell lines. Statistical significance and p-value determined using multiple unpaired t tests using Graphpad Prism (ns = not significant, * = $p < 0.05$).

A

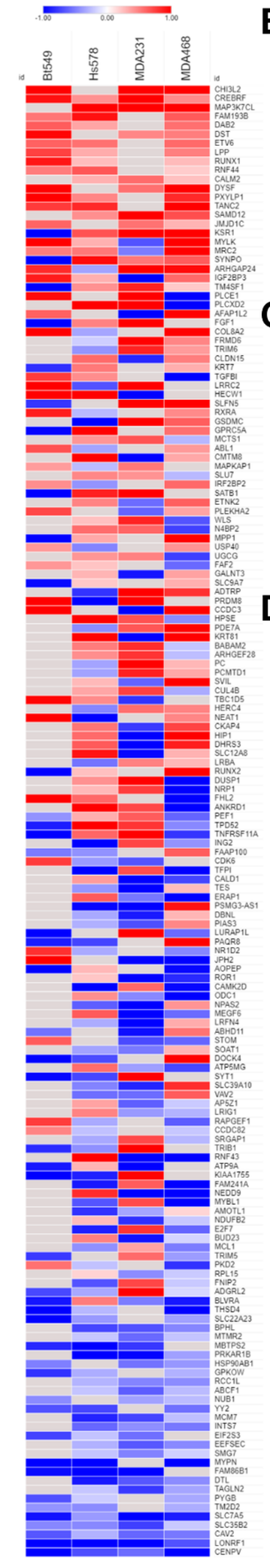

B

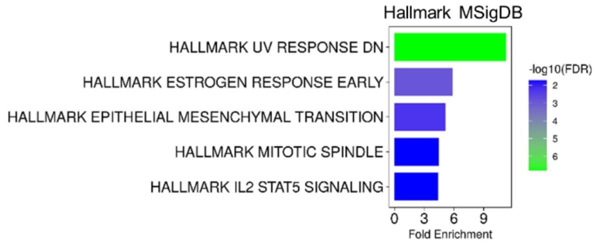

C

Core Targets of FOXC1 in TNBC

Ligands	Receptors	Transcription Factors	Kinases	LncRNA
TGFBI	WLS	ETV6	ABL1	PSMG3-AS1
FGF1	ROR1	RUNX1	MYLK	NEAT1
SLFN5	RXRA	RUNX2	CDK6	
CHI3L2	MRC2	E2F7	TRIB1	
	ADGRL2	NR1D2	KSR1	
	TNFRS11A			

D

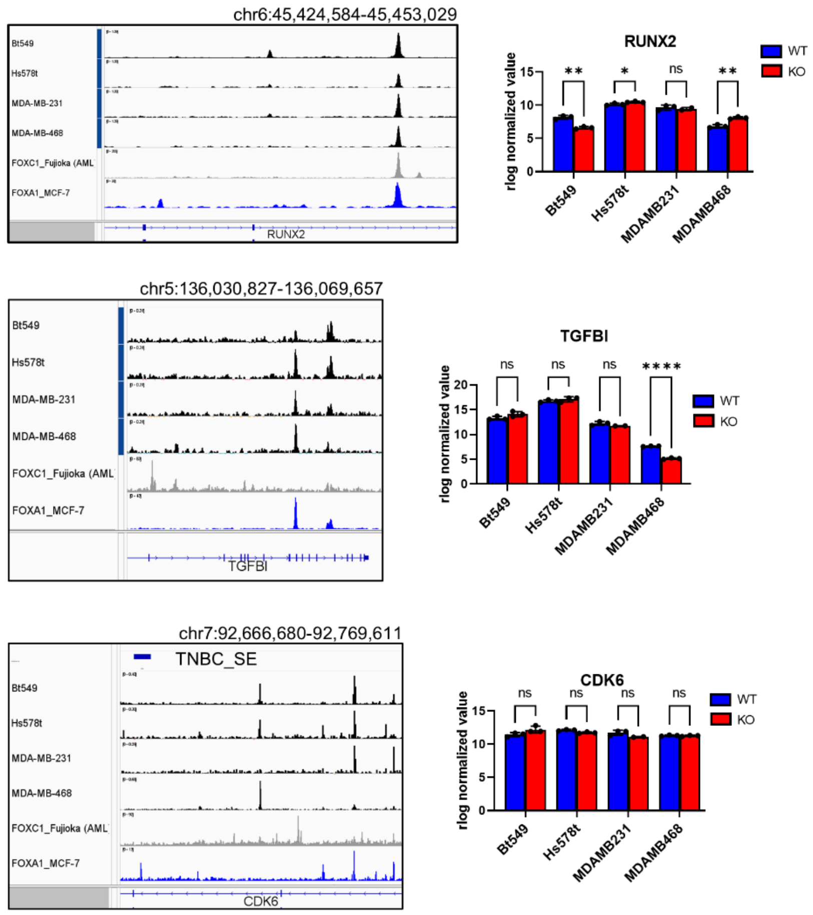

Fig. S9. Genes regulated by FOXC1 in TNBC as well as FOXA1 in luminal breast cancer (Related to Figure 5).

(A) Heatmap of log fold change (Log2FC) of normalized mRNA expression of the 164 core targets of FOXC1 in the four TNBC cell lines used in this study. **(B)** Enrichment of pathways from the hallmark gene set molecular signatures database (MSigDB) using the 164 core targets of FOXC1. **(C)** Selected genes from among the 164 core targets of FOXC1 that have an important role in cancer. **(D)** ChIP-seq traces and rlog normalized mRNA values from RNA-seq of FOXC1 core targets (RUNX2, TGFBI and CDK6) that have overlapping FOXA1 peaks in MCF-7. Statistical significance and p-value determined using multiple unpaired t tests using Graphpad Prism (ns = not significant, * = $p < 0.05$, ** = $p < 0.005$, **** = $p < 0.00005$).

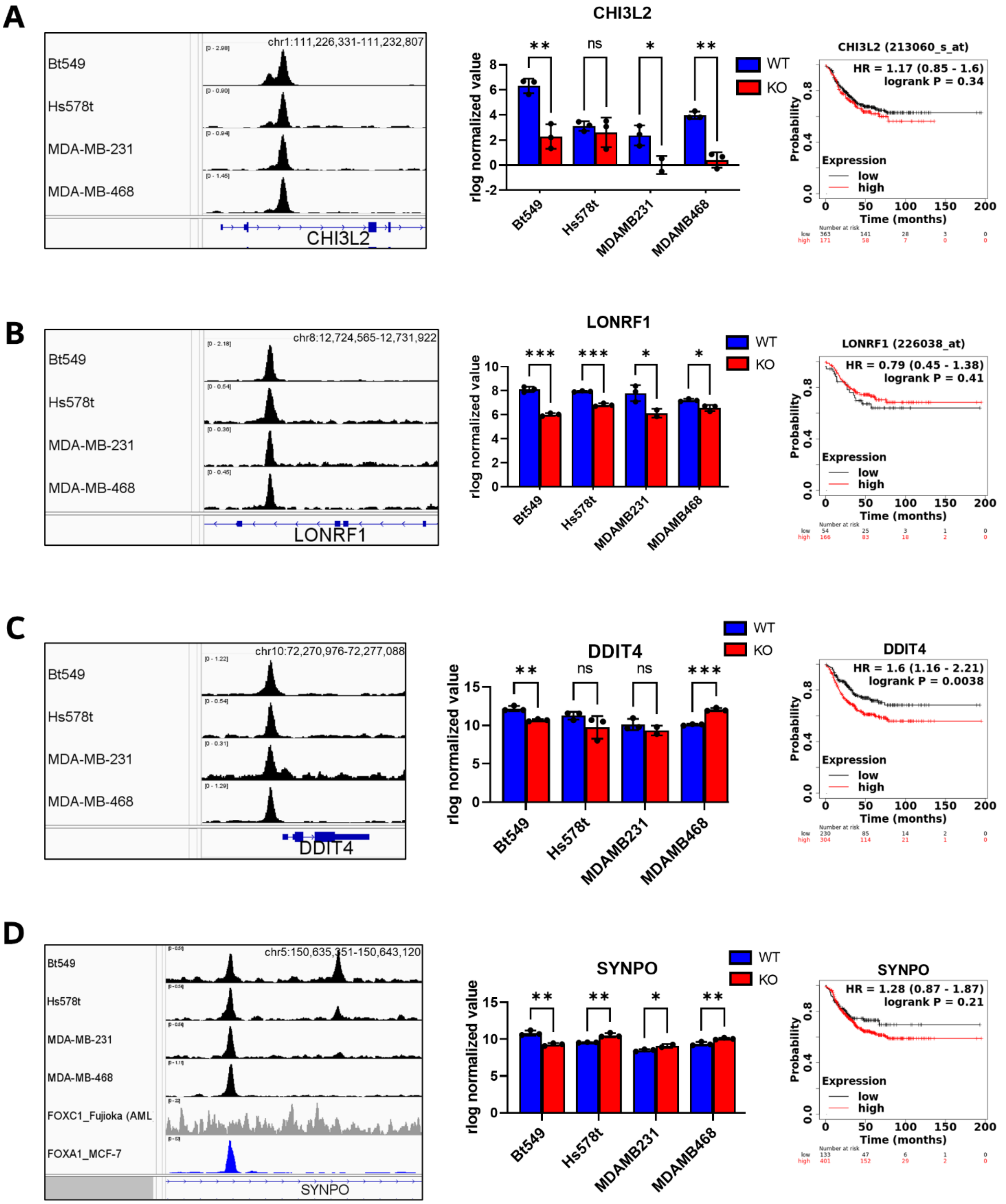

Fig. S10. Novel targets of FOXC1 (Related to Figure 5).

ChIP-seq traces and rlog normalized mRNA values from RNA-seq of FOXC1 core targets whose function is as yet unknown in TNBC **(A)** CHI3L2: FOXC1 binds the promoter and directly activates expression of CHI3L2 (Chitinase-3-Like Protein 2), and its expression strongly correlates with that of FOXC1 in patient tumor samples (Fig. 3f). Furthermore, the expression of CHI3L2 is associated with

poor outcomes in gliomas [11]. The function of this ligand in breast cancer is unknown, although CHI3L1, a closely associated ligand, is well-studied for its oncogenic role in many cancers [12]. **(B)** LONRF1: FOXC1 also activates expression of LONRF1 (LON Peptidase N-Terminal Domain and Ring Finger 1) via binding at intragenic sites, a binding site that is conserved in Fujioka cell line as well. Although the role of LONRF1 in breast cancer is yet to be studied, LONRF1 is downregulated in prostate cancer patient samples and is speculated to be part of the core set of genes for estimating recurrence and prognosis of prostate cancer [13]. **(C)** DDIT4: Another prognostic factor, DDIT4 (REDD1), is a key protein in cancer cachexia and hypoxia involved in Hif1 signalling whose expression correlates with chemoresistance in TNBC [14]. **(D)** SYNPO: SYNPO (Synaptopodin) is regulated by FOXA1 in MCF-7 as well. Although there are no studies for the role of SYNPO in breast cancer, SYNPO2, a related protein, is a well-studied oncogene that promotes metastasis in breast cancer via PI3K/Akt/mTOR pathway [15]. Bar plots show the normalised expression of mRNA in parental (Blue, WT) and FOXC1_KO (Red, KO) in TNBC cell lines. Statistical significance and p-value determined using multiple unpaired t tests using Graphpad Prism (ns = not significant, * = $p < 0.05$, ** = $p < 0.005$, *** = $p < 0.0005$).

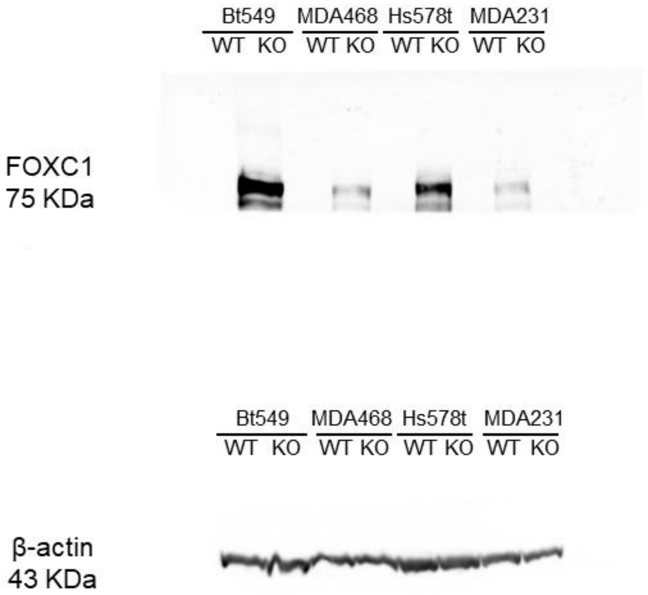

Data S1/Methods S1: Full uncropped Western Blot image, related to Figure 1A. Protein expression levels of FOXC1 and β-actin in four TNBC cell lines and their CRISPR FOXC1_KO clones (BT549, MDA-MB-468, Hs578t, MDA-MB-231). After transfer, the nitrocellulose membrane was cut into two strips, and the top strip was incubated with anti-FOXC1 (ab227977) and the bottom strip was incubated with anti-β-actin (ab6276). A total of 75 µg of protein was loaded to ensure no FOXC1 protein expression is visible in the FOXC1-KO clones.

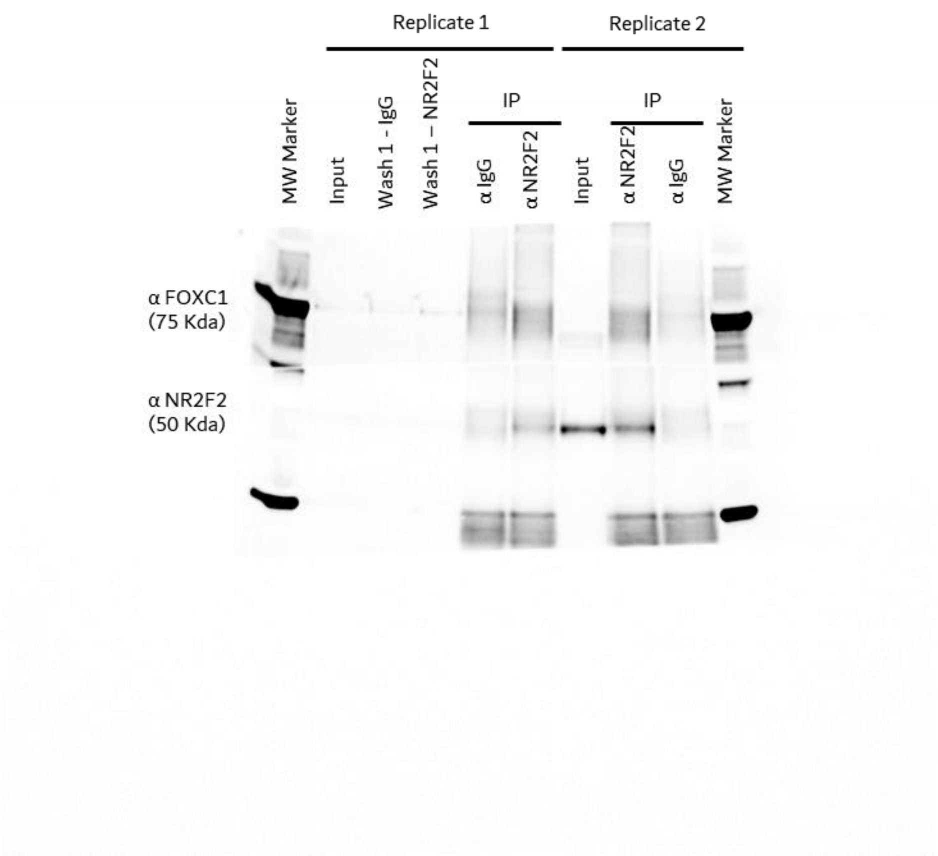

Data S2/Methods S2: Full uncropped Western Blot image, related to Figure 4B. Protein expression of FOXC1 and NR2F2 in co-immunoprecipitation assays performed as described in the Methods section. After transfer, the nitrocellulose membrane was cut into two strips, and the top strip was incubated with anti-FOXC1 (ab227977) and the bottom strip was incubated with anti-NR2F2 (ab211777). Molecular weight marker on the left-most and right-most lane shows 80 KDa (top band) and 30 KDa (bottom band).

Table S1: **Characteristics of the four TNBC cell lines chosen in this study [16] (Related to Figure 1).**

Name	Expression markers	Molecular classification /subtyping	Ethnicity	P53/BRCA1	PI3K Pathway	Other features	Rb1 expression [17]
BT-549	None	Mesenchymal/Basal B	Caucasian	Mut/WT	PTEN homo deletion		No RB1 expression
Hs578t	None	Mesenchymal-stem like/ Basal B	Caucasian	Mut/WT	Wt	HRas mutation	Normal phosphorylated forms of pRb
MDA-MB-231	EGFR, TGF	Mesenchymal-stem like/ Basal B	Caucasian	Mut/WT	Wt	KRas mutation	Normal phosphorylated forms of pRb
MDA-MB-468	EGFR, TGF	Basal-like 1/ Basal A	Black	Mut/WT	PTEN homo deletion	Amplified EGFR	No pRb expression

SUPPLEMENTARY REFERENCES

1. Simeoni, F., Romero-Camarero, I., Camera, F., Amaral, F.M.R., Sinclair, O.J., Papachristou, E.K., Spencer, G.J., Lie, A.L.M., Lacaud, G., Wiseman, D.H., et al. (2021). Enhancer recruitment of transcription repressors RUNX1 and TLE3 by mis-expressed FOXC1 blocks differentiation in acute myeloid leukemia. Cell Rep *36*, 109725. 10.1016/j.celrep.2021.109725.
2. Huang, H., Hu, J., Maryam, A., Huang, Q., Zhang, Y., Ramakrishnan, S., Li, J., Ma, H., Ma, V.W.S., Cheuk, W., et al. (2021). Defining super-enhancer landscape in triple-negative breast cancer by multiomic profiling. Nat Commun *12*, 2242. 10.1038/s41467-021-22445-0.
3. Jerzak, K.J., Cockburn, J.G., Dhesy-Thind, S.K., Pond, G.R., Pritchard, K.I., Nofech-Mozes, S., Sun, P., Narod, S.A., and Bane, A. (2018). Thyroid hormone receptor beta-1 expression in early breast cancer: a validation study. Breast Cancer Res Treat *171*, 709-717. 10.1007/s10549-018-4844-5.
4. Voutsadakis, I.A. (2022). The TSH/Thyroid Hormones Axis and Breast Cancer. J Clin Med *11*. 10.3390/jcm11030687.
5. Bolf, E.L., Gillis, N.E., Barnum, M.S., Beaudet, C.M., Yu, G.Y., Tomczak, J.A., Stein, J.L., Lian, J.B., Stein, G.S., and Carr, F.E. (2020). The Thyroid Hormone Receptor-RUNX2 Axis: A Novel Tumor Suppressive Pathway in Breast Cancer. Horm Cancer *11*, 34-41. 10.1007/s12672-019-00373-2.
6. Feng, M., Bao, Y., Li, Z., Li, J., Gong, M., Lam, S., Wang, J., Marzese, D.M., Donovan, N., Tan, E.Y., et al. (2014). RASAL2 activates RAC1 to promote triple-negative breast cancer progression. J Clin Invest *124*, 5291-5304. 10.1172/JCI76711.
7. Liu, H., Paddock, M.N., Wang, H., Murphy, C.J., Geck, R.C., Navarro, A.J., Wulf, G.M., Elemento, O., Haucke, V., Cantley, L.C., and Toker, A. (2020). The INPP4B Tumor Suppressor Modulates EGFR Trafficking and Promotes Triple-Negative Breast Cancer. Cancer Discov *10*, 1226-1239. 10.1158/2159-8290.CD-19-1262.
8. El Ansari, R., Craze, M.L., Miligy, I., Diez-Rodriguez, M., Nolan, C.C., Ellis, I.O., Rakha, E.A., and Green, A.R. (2018). The amino acid transporter SLC7A5 confers a poor prognosis in the highly proliferative breast cancer subtypes and is a key therapeutic target in luminal B tumours. Breast Cancer Res *20*, 21. 10.1186/s13058-018-0946-6.
9. Ren, J., Chen, Y., Kong, W., Li, Y., and Lu, F. (2021). Tumor protein D52 promotes breast cancer proliferation and migration via the long non-coding RNA NEAT1/microRNA-218-5p axis. Ann Transl Med *9*, 1008. 10.21037/atm-21-2668.
10. Lawrence, R.T., Perez, E.M., Hernandez, D., Miller, C.P., Haas, K.M., Irie, H.Y., Lee, S.I., Blau, C.A., and Villen, J. (2015). The Proteomic Landscape of Triple-Negative Breast Cancer. Cell Rep *11*, 990. 10.1016/j.celrep.2015.04.059.
11. Liu, L., Yang, Y., Duan, H., He, J., Sun, L., Hu, W., and Zeng, J. (2021). CHI3L2 Is a Novel Prognostic Biomarker and Correlated With Immune Infiltrates in Gliomas. Front Oncol *11*, 611038. 10.3389/fonc.2021.611038.
12. Zhao, T., Su, Z., Li, Y., Zhang, X., and You, Q. (2020). Chitinase-3 like-protein-1 function and its role in diseases. Signal Transduct Target Ther *5*, 201. 10.1038/s41392-020-00303-7.
13. Guo, H., Zhang, Z., Wang, Y., and Xue, S. (2021). Identification of crucial genes and pathways associated with prostate cancer in multiple databases. J Int Med Res *49*, 3000605211016624. 10.1177/03000605211016624.

14. Horak, P., Crawford, A.R., Vadysirisack, D.D., Nash, Z.M., DeYoung, M.P., Sgroi, D., and Ellisen, L.W. (2010). Negative feedback control of HIF-1 through REDD1-regulated ROS suppresses tumorigenesis. Proc Natl Acad Sci U S A *107*, 4675-4680. 10.1073/pnas.0907705107.
15. Xia, E., Zhou, X., Bhandari, A., Zhang, X., and Wang, O. (2018). Synaptopodin-2 plays an important role in the metastasis of breast cancer via PI3K/Akt/mTOR pathway. Cancer Manag Res *10*, 1575-1583. 10.2147/CMAR.S162670.
16. Chavez, K.J., Garimella, S.V., and Lipkowitz, S. (2010). Triple negative breast cancer cell lines: one tool in the search for better treatment of triple negative breast cancer. Breast Dis *32*, 35-48. 10.3233/BD-2010-0307.
17. Robinson, T.J., Liu, J.C., Vizeacoumar, F., Sun, T., Maclean, N., Egan, S.E., Schimmer, A.D., Datti, A., and Zacksenhaus, E. (2013). RB1 status in triple negative breast cancer cells dictates response to radiation treatment and selective therapeutic drugs. PLoS One *8*, e78641. 10.1371/journal.pone.0078641.
